# Epistemic planning for multi-robot systems in communication-restricted environments

**DOI:** 10.3389/frobt.2023.1149439

**Published:** 2023-05-23

**Authors:** Lauren Bramblett, Nicola Bezzo

**Affiliations:** ^1^ Autonomous Mobile Robots Lab, Link Lab, Department of Systems and Information Engineering, University of Virginia, Charlottesville, VA, United States; ^2^ Autonomous Mobile Robots Lab, Link Lab, Department of Electrical and Computer Engineering, University of Virginia, Charlottesville, VA, United States

**Keywords:** multi-robot system, epistemic planning, task allocation, swarming, planning under intermittent communication, cooperative planning and control

## Abstract

Many real-world robotic applications such as search and rescue, disaster relief, and inspection operations are often set in unstructured environments with a restricted or unreliable communication infrastructure. In such environments, a multi-robot system must either be deployed to i) remain constantly connected, hence sacrificing operational efficiency or ii) allow disconnections considering when and how to regroup. In communication-restricted environments, we insist that the latter approach is desired to achieve a robust and predictable method for cooperative planning. One of the main challenges in achieving this goal is that optimal planning in partially unknown environments without communication requires an intractable sequence of possibilities. To solve this problem, we propose a novel epistemic planning approach for propagating beliefs about the system’s states during communication loss to ensure cooperative operations. Typically used for discrete multi-player games or natural language processing, epistemic planning is a powerful representation of reasoning through events, actions, and belief revisions, given new information. Most robot applications use traditional planning to interact with their immediate environment and only consider knowledge of their own state. By including an epistemic notion in planning, a robot may enact depth-of-reasoning about the system’s state, analyzing its beliefs about each robot in the system. In this method, a set of possible beliefs about other robots in the system are propagated using a Frontier-based planner to accomplish the coverage objective. As disconnections occur, each robot tracks beliefs about the system state and reasons about multiple objectives: i) coverage of the environment, ii) dissemination of new observations, and iii) possible information sharing from other robots. A task allocation optimization algorithm with gossip protocol is used in conjunction with the epistemic planning mechanism to locally optimize all three objectives, considering that in a partially unknown environment, the belief propagation may not be safe or possible to follow and that another robot may be attempting an information relay using the belief state. Results indicate that our framework performs better than the standard solution for communication restrictions and even shows similar performance to simulations with no communication limitations. Extensive experiments provide evidence of the framework’s performance in real-world scenarios.

## 1 Introduction

Multi-robot systems (MRSs) have the potential to improve efficiency, flexibility, and scalability in various tasks. However, coordinating cooperation for multiple robots can be a challenging problem, particularly in dynamic and uncertain environments with limited communication. In application spaces where long-range communication is often unreliable or unavailable, we observed a current limitation in MRS research where most approaches assume constant information sharing between robots (Hussein et al., 2014; Liu et al., 2022). Generally, MRS applications with communication constraints are high-stake scenarios such as finding a stranded hiker in a remote location, recovering pieces of a downed aircraft in hostile territory, or rescuing survivors after a natural disaster. Additionally, MRSs have been applied to scenarios with limited communication infrastructure such as subterranean pipeline inspection, marine sample collection, or extra-planetary exploration, where range, terrain, and environment can inhibit signals from being sent or received by any entity (Yliniemi et al., 2014; Manjanna et al., 2018; Kuang-wei et al., 2020). As humans, we cope with these constraints by implicitly reasoning about other actors’ actions or beliefs while not communicating. A person may empathize with what another actor might believe in order to communicate and come to a shared understanding of the environment as demonstrated in the Sally–Anne test (Baron-Cohen et al., 1985) and (Krupenye et al., 2016). In this work, we propose a similar construct, taking advantage of local observations and constructing a framework for robots to plan and communicate according to a set of higher-order beliefs while disconnected.

In our previous work (Bramblett et al., 2023), we presented a robust, failure-tolerant framework based on *epistemic planning* to formalize logical planning considering knowledge and beliefs of the MRS. This method allows for a distributed system to iteratively reason about the location of other robots in the system and behave according to that belief. Beliefs and knowledge were updated using a static time rendezvous, creating inefficiencies if the environment is known or only small deviations from any plan are required while disconnected. Within this framework, only tasks requiring one robot were considered, and each robot was able to accomplish tasks while disconnected, considering reconnection only when triggered by the artificial potential field.

We build on these ideas and formalize a problem in which the goal is to cooperatively explore, find, and accomplish tasks in the environment; however, the scenario is further complicated by tasks at unknown locations that may require multiple agents (e.g., lifting a heavy object or inspecting a large structure). Since the locations of these tasks are initially unknown, calculating a distributed plan for coverage while accounting for any combination of a robot system’s actions, changes in the environment, or deviations is intractable over long periods of disconnection. Alternatively, establishing a reasoning framework for a finite set of possibilities for each robot can reduce computational complexity and increase the mission efficiency. Thus, we introduce an epistemic prediction and planning method with gossip protocol in which a robot propagates a finite set of *belief* states representing possible states of other agents in the system and *empathy* states representing a finite set of possible states from other agents’ perspectives. Each agent may attempt to communicate and allocate found tasks by traveling to the believed location of another agent.

**FIGURE 1:**
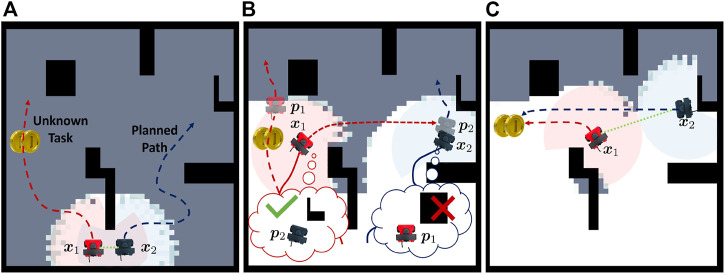
Pictorial depiction of the problem presented in this paper. The proposed framework enables a robot to reason from other robots’ perspectives as it experiences a behavior change or observes that another robot is not where expected. **(A)** shows the initial plan. **(B)** shows robot 1 finding a task that requires robot 2 and routing to robot 2’s belief state. **(C)** shows robot 1 and robot 2 communicating and routing to accomplish the task.

Consider Figure 1 where two robots are canvassing an environment. During disconnection, robot 1 maintains a set of belief states for robot 2 (***p***_2_) and also a set of empathy states that robot 2 might believe about robot 1 (***p***_1_). Once robot 1 finds a task that also requires robot 2, it attempts to communicate by routing to robot 2’s belief state shown in Figure 1B. Robot 1 travels to the believed location of robot 2 and is able to communicate if ***p***_2_ is a close approximation of ***x***_2_, illustrated in Figure 1C. We reason that though robot 2 holds a false belief about robot 1’s state, there exists an epistemic strategy that allows robot 1 to communicate with robot 2 (i.e., robot 1 propagating and checking the belief state for robot 2 and by robot 2 empathizing with robot 1’s belief).

The contribution of our approach is two-fold: i) an epistemic planning and propagation formulation using dynamic epistemic logic, formalizing beliefs, and knowledge for consensus-based coverage while disconnected and ii) a generalized consensus-based epistemic task assignment and gossiping protocol for multi-robot tasks with considerations for connectivity constraints and team member dynamics.

The rest of the paper is organized as follows: Section 2 provides an overview of the current research in multi-robot coverage, task allocation, and epistemic planning. Section 3 explains assumptions for communication and control while introducing the fundamentals of dynamic epistemic logic. Section 4 formally defines the problem, followed by the framework for belief propagation, coverage, epistemic planning, and task allocation in Section 5. Simulations and experiments validating our method are presented in Section 6 and Section 7, respectively. Finally, the conclusion and future work are discussed in Section 8.

## 2 Background

Multi-robot exploration, foraging, and coverage remain an open problem in robotics literature (Zhou et al., 2019; Kwa et al., 2022; Poudel and Moh, 2022). Recent works have expanded the problem formulation to consider communication restrictions or intermittent connectivity by modeling ways to maintain connection while exploring (Capelli and Sabattini, 2020), accounting for intermittent communication (Best et al., 2018), or by placing markers where other robots have already explored (Cardona et al., 2019). Matignon et al. (2012) used a decentralized Markov decision process to predict the MRS future state for stochastically delayed messages.

A related field of multi-robot research is multi-robot task allocation (MRTA). MRTA assigns a subset of robots to a variety of tasks to complete a global objective (Kim et al., 2020). The overarching taxonomy and various solution techniques are described by Korsah et al. (2013); however, most of these algorithms focus on solutions, given perfect and complete information. Chen et al. (2022) included connection limitations and allocated tasks using a consensus-based bundling algorithm (CBBA) with robots within communication range, but assumed dynamic tasks can be accomplished by the local team. Additionally, Schoenig and Pagnucco (2010) used sequential-single item (SSI) auctions for dynamic tasks, comparing different schemas for evaluating task allocation when all tasks are not initially known.

Though recent works have included realistic constraints that mirror real-world operations for coverage and task allocation, there is little consideration for the combination of prolonged disconnection with task discovery and allocation. Otte et al. (2020) tackled a similar problem using an auction allocation algorithm to assign tasks in a communication-limited environment, but it is assumed that the number of robots present in the local connected network is adequate to complete the discovered tasks. In our previous work (Bramblett et al., 2022), we defined rendezvous points at known locations to coordinate roles for any events during exploration; however, we noticed robots back-tracking to a predefined location reduced efficiency of exploration. Instead, to decrease the need for unnecessary communication or laborious rendezvous, this work applies dynamic epistemic logic (DEL) (Van Ditmarsch et al., 2007) to allow a robot to reason about higher-order beliefs among actors in a multi-robot system while disconnected and allocate tasks with limited communication. DEL is typically used in game theory applications to describe knowledge and belief shifts for players in a game, but recently has been integrated in robotics applications. Using robot and human actors, the framework presented by Bolander et al. (2021) recreates the Sally–Anne psychological test where a robot must reason about the human’s beliefs. Moreover, DEL has been used to solve for cooperative actions in multi-player games with implementation on a multi-robot system, (Maubert et al., 2021). In this work, we use DEL to allow robots to reason, given their respective knowledge and beliefs, about the system’s state considering task discovery, communication requirements, and partially unknown environments.

## 3 Preliminaries

### 3.1 Notation, communication, and control

Let us consider a multi-robot system of *N*_*r*_ robots in the set A . We note that initial positions of the robots are known. The system’s connectivity graph is denoted as G=(A,E) , where the set E⊂A×A represents edge connections between robots. An edge (i,j)∈E indicates that robots *i* and *j* are within the communication range (i.e., connected). For ease, motivated by most wireless communication modules with a limited range such as Wi-Fi, LoRa, and Bluetooth, we abstract communication range as a disk centered on the robot. Robots *i* and *j* are considered connected if they are within the communication range, *r*_*c*_.

Additionally, a number of tasks *N*_*t*_ in the set T are located in unknown positions within the operating environment. Initially, *N*_*t*_ may be known or unknown. An element *τ* in T is defined by the tuple identifying the location, number of required robots, and reward: (*x*_*τ*_, *y*_*τ*_, *r*_*τ*_, *λ*_*τ*_). We assume the tasks are stationary and completed once a subset of robots navigate within a radius *r*_*t*_ > 0.

The robots are assigned to search for the tasks in an environment, W , that is partitioned into *N*_*m*_ cells, which we define as an occupancy map $M\subseteq R^{2}$ . When robots navigate to observe unexplored cells $M_{u}\subseteq M$ , M is updated using recursive Bayesian estimation (Asgharivaskasi and Atanasov, 2021), though any method can be used. Subsequently, we define the Frontier set $F\subseteq M\backslash M_{u}$ as the set of explored cells adjacent to unknown cells. We assume that the entirety of the exploration area is partially unknown.

Without loss of generality, each of the robots is modeled as a linear time-invariant (LTI) dynamical agent such that

$${\dot{x}}_{i}=Ax_{i}+Bu_{i}+\nu_{i},\;\forall i\in A,$$ (1)

where $x_{i}\in R^{n}$ is the robot *i*’s state vector, $u_{i}\in R^{m}$ is the control input, and ***A*** and ***B*** are state and input matrices, respectively. The variable $\nu_{i}\in R^{n}$ denotes zero-mean Gaussian process uncertainty. We let a state of robot *i*, ***x***_*i*_, represent not only the location and dynamics of the robot but also its local occupancy map and status. *Status* is defined as a robot’s current objective such as covering the environment, communicating, or completing a task. We let robot *i*’s status be denoted as proposition *σ*_*i*_ and represents which objective a robot is executing.

### 3.2 Epistemic logic

In this work, epistemic and doxastic logic (Rendsvig and Symons, 2019) is used to model distributed knowledge and reasoning for system changes during disconnectivity. We define an epistemic state with the following definition.

Definition 1. *An epistemic state is classically described using a tuple* *s* = (*W*, *R*_*i*_, *V*, *W*_*d*_) *for a countable set of atomic propositions,* *AP* *, where*

• *W* *is a non-empty, finite set of possible worlds.*
• *R*_*i*_ ⊆ *W* × *W* *is an accessibility relation for robot* *i.*
• *V* → 2^*AP*^ *is a valuation function.*
• *W*_*d*_ ⊆ *W* *is the set of designated worlds from which all worlds in* *W* *are reachable.*

The formula *vR*_*i*_ *w* means that though the actual world is *w*, robot i∈A believes the world is *v*. We also define *s* as the epistemic state and set the initial epistemic state to *s*_0_ = (*W*, *R*, *V*, *w*_0_), where *W*_*d*_ = {*w*_0_} means that *s*_0_ is the global epistemic state. A world, *w*, signifies the set of true propositions, which in our application is the status of each robot $w=\left\{\sigma_{i}\forall i\in A\right\}$ .

To propagate the states of robots, we define beliefs as the set of estimated locations of all robots in the system from each robot’s perspective. The set $P=\left\{P_{1},\ldots ,P_{N_{a}}\right\}$ holds the distributed beliefs of all agents, where an element in $P_{i}$ represents possible states from agent *i*’s perspective of robots j∈A . Ψ is a set of functions that describe the current state of the system. For this application, the epistemic language, L(Ψ,P,A) , is obtained as follows in the Backus–Naur form (Knuth, 1964):

$$\phi ⩴H\left(\omega \right)\;|\;\phi \wedge \phi \;|\;\neg \phi \;|\;K_{i}\phi \;|\;B_{i}\phi ,$$

where i,j∈A , *H* ∈ Ψ is a function to describe a system state, and *ω* broadly indicates function arguments. *¬ϕ* and *ϕ* ∧ *ϕ* denote that propositions can be negated and form logical conjunctions, respectively. *B*_*i*_ *ϕ* and *K*_*i*_ *ϕ* are interpreted as “agent *i* believes *ϕ*” and “agent *i* knows *ϕ*, respectively.”

Dynamic epistemic logic is expanded from epistemic logic through action models. These models affect how robots perceive an event and its effects on the world.

Definition 2. *An action model* $L=\left(A,R_{i}^{L},pre,post\right)$ *is a tuple with the following definitions*

• *A* *is a non-empty, finite set of possible actions.*
• $R_{i}^{L}\subseteq A\times A$ *is an accessibility relation for agent* *i* *in the action model.*
• *pre* *is a precondition for an action to be performed.*
• *post* *is a post-condition or effects of an action.*

As such, the epistemic product model is formally introduced as $s⊗i:\;a=\left(W^{\prime },R_{i}^{\prime },V^{\prime },W_{d}^{\prime }\right)$ , where *i*: *a* indicates that an action *a* has been executed by robot *i*. In this paper, we describe a robot’s main actions that can occur as follows: *perceive* a robot or task and *announce* a proposition or system state. The worlds that the system can be in are described by the combinations of all possible statuses of each robot in the multi-robot system.

## 4 Problem formulation

In this paper, we consider a scenario in which a multi-robot system must coordinate in a decentralized fashion to efficiently search for tasks at unknown locations in a communication-restricted, partially unknown environment. We focus on a subcategory of the MRTA problems known as the single-task, multi-robot, time-extended allocation problem [ST-MR-TA], meaning that each robot can only execute one task at a time, and tasks may require multiple robots. There are several challenges that arise to allow efficient and cooperative behavior, given limited communication, including 1) how to efficiently cover a partially unknown environment for tasks; 2) upon discovery, how should tasks be ideally allocated to a subset of robots; and 3) how to communicate necessary information to robots in the system if disconnected. Formally, we define these problems as follows:

**Problem 1 (*Communication-restricted coverage*):** Find a distributed policy to enable a multi-robot system to quickly perform distributed and cooperative coverage of a partially unknown environment with intermittent communication. The robots should safely navigate the environment, given a set of unknown obstacles that may cause the robot to deviate from an original plan.

**Problem 2 (*Communication-restricted task allocation*):** Find a distributed policy to enable a multi-robot system to dynamically solve a task allocation problem given that an allocation may necessitate communication with a subset of disconnected robots.

To solve **Problem 1**, we propose an epistemic planning approach that consists of two main parts. First, we propagate a set of global (common) belief states that inform the approximate location of a robot cooperatively covering the environment. Then, we use a common belief set to partition the environment for coverage, which informs how belief particles are propagated in the next iteration. To solve **Problem 2**, we propose a decentralized task allocation algorithm that assigns robots to discovered tasks and communication responsibilities.

## 5 Approach

**FIGURE 2:**
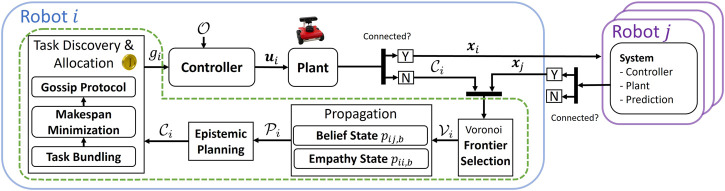
Diagram of the proposed approach. The contributions of this work are shown within the green box.

In this section, we present the approach for the coordinated epistemic prediction, planning, and allocation framework which propagates belief and empathy states to inform Frontier assignment and robot control, all while considering task discovery and unknown obstacles. For ease of discussion, let us consider two robots *i* and *j*. From robot *i*’s perspective, a *belief state*, $p_{ii,j}\in P_{i}$ , represents a possible state of robot *j* and an *empathy state*, $p_{ij,i}\in P_{i}$ , describes robot *i*’s belief of robot *j*’s belief about robot *i*’s state. Once robots *i* and *j* disconnect, robot *i* holds a main belief about robot *j* and empathizes with what robot *j* might believe, which we label as the common belief set, $C_{i}$ . With this knowledge, robot *i* predicts and tracks empathy states to maintain robot *j*’s belief of the state of robot *i*. The common belief, $C_{i}$ , is used for decentralized planning if the robots have no updates for long periods of disconnection. The diagram in Figure 2 summarizes this proposed architecture.

Coverage is accomplished via a cooperative multi-robot Frontier-based method due to its simplicity, completeness, and efficiency (Fox et al., 2006). As shown in Figure 2, the robot *i* initially assesses whether communication is successful with a robot *j*. As we will discuss in this section, if communication is successful, robot *i* uses its current state ***x***_*i*_ and the state of robot *j*, ***x***_*j*_, to compute the Voronoi diagram, $V_{i}$ (Cortes et al., 2004). The Voronoi diagram informs a robot’s utility for traveling to any Frontier point, and the robot selects the Frontier point with the highest utility, *g*_*i*_. Travel to this goal point is conducted via a smooth A* planner and artificial potential field (APF), though any applicable method can be used. When connected, epistemic planning is reduced to direct communication of states. If the robots disconnect, the common belief set, $C_{i}$ , acts as the state for any robot j∈A from *i*’s perspective. Predictions for these belief and empathy states are accomplished using the same Voronoi partitioning and path planning methods. Robot *i* then uses these predicted states to plan considering its belief about robot *j*.

In both connected and disconnected conditions, the robot’s objective is to search for tasks. If connected and a task is discovered, the robots bid on and allocate the discovered tasks. If disconnected or if enough robots are not present at the task, a single robot will submit bids on behalf of other robots using its belief states.

In the following sections, we lay out the key components of the planner including i) belief and empathy propagation, ii) coverage assignments for disconnected robots, iii) epistemic planning for belief consensus, and iv) epistemic task allocation.

### 5.1 Belief and empathy propagation

In our coordinated epistemic prediction, planning, and allocation framework, the robots propagate belief and empathy states for all robots in the multi-robot system. This allows robot *i* to plan according to its belief of other robots and reason about what other robots expect robot *i* to accomplish while disconnected. As previously noted, to account for uncertainties over long periods of disconnection, it is important to have a finite number of these states. With this goal in mind, we define a finite set of particles, $P_{i}$ , to represent these belief and empathy states for the *i*th robot:

$$P_{i}=\left\{p_{ij,k}\;\forall j\in A,\forall k\in A\right\}.$$ (2)

The *i*th robot defines its empathy particles as $P_{i}^{e}=\left\{p_{ij,i}\;\forall j\in A\right\}$ and its belief particles about other robots as $P_{i}^{r}=\left\{p_{ij,k}\;\forall j\in A,\forall k\in A⧵\left\{i\right\}\right\}$ , where $P_{i}=P_{i}^{e}\cup P_{i}^{r}$ . The particle *p*_*ij*,*k*_ is interpreted as a second-order belief (a belief about beliefs) and represents robot *i*’s belief about robot *j*’s belief about robot *k*’s state. To start, all particles are set as the robots’ initial state.

While not in the communication range of other robots, each robot *i* propagates a subset of belief particles from the last globally communicated state between robot *i* and robot *j*. We define this set of particles as $C_{i}\subseteq P_{i}$ and refer to it as robot *i*’s *common belief* set. All robots track a second-order belief or empathy particle, *p*_*ij*,*i*_, upon disconnection, whose motion is planned using the common belief set, $C_{i}=\left\{c_{ij}\;\forall j\in A\right\}$ . Each particle $c_{ij}\in C_{i}$ propagates according to the last global epistemic state. The common belief is reset when all robots are within the communication range and new knowledge is shared (i.e., coverage, unknown obstacles, and tasks).

Each particle, *p*_*ij*,*k*_, is propagated toward its goal state, *g*_*ij*,*k*_, using the given vehicle dynamics and a smoothed A* path planning algorithm (Mueggler et al., 2014). The goal selection is dependent on a particle’s status. Within this paper, there are four main statuses that each particle can be in: *exploring*, *gossiping*, *completing a goal*, or *going home*, observing that these statuses are predefined and mission-dependent. The go-to-goal behavior for each particle is accomplished via an artificial potential field (APF) (Khatib, 1986) because of its simplicity and calculation speed. When the APF is coupled with the A* path planning algorithm, local minima are avoided. The APF construct formulates a repulsive force around threats such as obstacles and other robots, *F*_*rep*_, and an attractive force, *F*_*att*_, toward the goal. The composite potential field for these forces is formed by the following equations for a generic particle, *p*.

$$U_{\mathrm{att}}=\frac{1}{2}\eta_{g}\rho_{g}^{2}\left(p\right),$$ (3)

$$U_{\mathrm{rep}}=\left\{\begin{array}{cc} \frac{1}{2}\eta_{o}{\left(\frac{1}{\rho \left(p\right)}-\frac{1}{\rho_{o}}\right)}^{2},\; & \rho \left(p\right)\leq \rho_{o}\\ 0,\; & \rho \left(p\right)>\rho_{o} \end{array}\right.,$$ (4)

$$U_{\mathrm{total}}=U_{\mathrm{att}}+U_{\mathrm{rep}},$$ (5)

where *ρ*_*g*_ and *ρ* are the distance functions from the target and threats, respectively, with *η*_*g*_ and *η*_*o*_ representing the gain coefficients for attraction and repulsion, respectively. The subsequent composite forces that govern the particles’ motion are.

$$F_{\mathrm{att}}=\eta_{g}\rho_{g}\left(p\right),$$ (6)

$$F_{\mathrm{rep}}=\left\{\begin{array}{cc} -\eta_{o}\left(\frac{1}{\rho \left(p\right)}-\frac{1}{\rho_{o}}\right){\left(\frac{1}{\rho \left(p\right)}\right)}^{2}▿\rho \left(p\right),\; & \rho \left(p\right)\leq \rho_{o}\\ 0,\; & \rho \left(p\right)>\rho_{o} \end{array}\right.,$$ (7)

$$F_{\mathrm{total}}=F_{\mathrm{att}}+F_{\mathrm{rep}},$$ (8)

where ▿ denotes the gradient.

A robot tracks an empathy particle, considering unknown obstacles may cause deviations in the predicted path. Since the robot will be tracking an empathy particle, particle propagation must encourage efficient coverage of the environment. Thus, we introduce an epistemic Frontier-based coverage algorithm to motivate motion toward distinct, uncovered regions of the environment while disconnected.

### 5.2 Epistemic coverage assignments

The majority of distributed coverage algorithms depend on either a globally connected network or a limited, asynchronous communication within a small, finite amount of time (El Shenawy et al., 2020; Hu et al., 2020). Many overcome this limitation by simply choosing the closest Frontier point to a robot (Cesare et al., 2015) or retaining the last position of the robot and only sharing information if the robots wander within range (Colares and Chaimowicz, 2016). In contrast, we introduce a partitioning and coverage mechanism using the common belief set, C , for cooperative robots, given a partially unknown environment while disconnected.

To begin, each robot updates its true local map using recursive Bayesian estimation (Asgharivaskasi and Atanasov, 2021). Each robot also simulates updates for each *j* ^th^ particle in the set $P_{i}$ with robot *j*’s respective sensor parameters. Using the common belief particles in set $C_{i}$ and their corresponding maps, each robot determines its Frontier set, $F_{i}$ , by assessing which explored cells are adjacent to unknown cells. Additionally, the optimal partition of $F_{i}$ is the Voronoi partition $V_{i}\left(C_{i}\right)=\left\{V_{i1},V_{i2},\ldots ,V_{iN_{r}}\right\}$ generated by common belief particles in $C_{i}$ denoted as the points $\left(c_{i1},c_{i2},\ldots ,c_{iN_{r}}\right)$ :

$$V_{ij}=\left\{f\in F_{i}|\;\;\|f-c_{ij}\|\leq \|f-c_{ik}\|,\forall j\neq k\right\}.$$ (9)

Using the common belief set versus the communicated location of robots allows for decentralized coverage while disconnected by implicitly reasoning about the assignments of other robots and their individual motion plans.

After determining each common belief particles’ Frontier partition, the utility of each Frontier point is assessed. The utility of a Frontier point is user-defined (e.g., distance to Frontier point, distance to other robots, and heading difference) while incorporating a penalty for Frontier points outside of a particles’ partition such that the utility of each Frontier point is defined as follows

$$\upsilon_{ij,z}=\left\{\begin{array}{cc} u\left(f_{z},\alpha_{j}\right)+\Delta \; & f_{z}\notin V_{ij}\\ u\left(f_{z},\alpha_{j}\right)\; & f_{z}\in V_{ij} \end{array}\right.,$$ (10)

where Δ is a penalty for Frontier points outside of a particles’ partition and *u*(⋅) is the utility function for assigning *c*_*ij*_ to $f\in F_{i}$ . Subsequently, the Frontier point that minimizes the utility from (10) is defined as

$$z^{*}=\underset{z}{\mathrm{argmin}}\;\upsilon_{ij,z},$$ (11)

and

$$g_{ij}^{c}=f_{z^{*}}.$$ (12)

**FIGURE 3:**
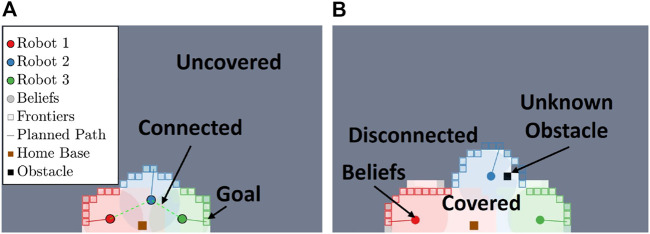
This figure shows the initial stages of coverage for three robots using the proposed epistemic coverage method. **(A)** shows three robots connected and partitioning the environment based on known states. **(B)** shows coverage using the epistemic belief to allocate frontiers in the environment. The actual coverage accomplished by each robot is represented by the light shaded region.

The variable $g_{ij}^{c}$ is the Frontier point goal for the common belief particle, *c*_*ij*_, which encourages the common belief to propagate toward unique, uncovered portions of the environment. If a particle’s status is *exploring*, it also shares the same goal as its respective common belief particle: $g_{ij,j}=g_{ij}^{c}\forall j\in A$ . Otherwise, the goal for each particle depends on the particle’s status such as going to a task, communicating with another robot, or traveling to home base. Figure 3 shows an example of particles propagating in a partially unknown environment. As shown in Figure 3A, the robots begin in the communication range and establish goals along the Frontier using (10). Figure 3B shows the robots disconnect as they move toward their respective Frontier goals and establish belief states. The plotted belief states for an *i* ^th^ robot are the belief states of all other robots and an empathy state from robot *i*’s perspective. The covered area is shaded by the robot color that accomplished coverage, and the plotted Frontier points are the Frontier points from each belief states’ perspective, dynamically allocated using (9). As the robot is traveling, unknown obstacles may appear, and the robot avoids these obstacles while continuing to follow its main empathy particle.

### 5.3 Epistemic updates and planning

Epistemic planning is a modal representation of planning about knowledge and beliefs when the environment changes. Under the assumption that robots have limited communication capabilities, the problem we are solving can be considered a game with imperfect information. Maubert et al. (2019) pointed out that multi-player games with imperfect information are undecidable, but using epistemic planning and assuming cooperative robots, we can tame the complexity of the problem to achieve consensus in most disconnected scenarios.

### 5.3.1 Epistemic update logic

Belief update is the process of accepting new information that may contradict initial beliefs. When robots communicate, any necessary belief updates must take place rationally to ensure global consensus is still retained. Thus, there are four cases in which belief update occurs in this work: i) when globally connected to all robots, ii) when locally connected to another robot, iii) when expecting to connect with another robot, and iv) upon task discovery. Referring to the previously established semantics for DEL in Section 3, we introduce our action library *A* that can transform the epistemic state. We let *A* = {*perceive*(*ϕ*), *announce*(*ϕ*)}. The action *perceive* is when a robot perceives a generic proposition *ϕ* in the environment, such as a task or robot, and the action *announce* is when a robot communicates with its locally connected team. Also, we introduce the set Ψ with one element such that Ψ = {*present*}, which is interpreted functionally in our application for *K*_*i*_ *present*(*τ*) as robot *i* knows the location, required robots, and value of task *τ*.

The global belief update is relatively simple. All new information is centrally known, so all particle states can be updated to known robot states instantaneously. We assume because robots are cooperative, all belief updates are accepted and do not become outdated unless an event occurs in the environment such as discovering a task; however, each robot may not know when/if the information of the system becomes outdated when disconnected. We formulate the logic for this framework using a series of worlds, *w*_*t*_, which is the set of propositions of each robot’s status, $\sigma_{i}^{t}\;\forall i\in A$ . Additionally, there exists one true world, $w_{t}^{*}$ , at time *t* and only exists if

$$w_{t}^{*}R_{i}w_{t}^{*},\;\forall i\in A.$$ (13)

In order for all robots to know with certainty the true world, all robots’ states, $\sigma_{t}^{i}\in w_{t}^{*}$ , must be common knowledge and announced such that the epistemic state from robot *i*’s perspective at time *t* is

$$s_{t-1}^{i}⊗announce\left(x\right)=s_{t}^{i}⊧K_{i}\sigma_{t}^{i}\underset{j\in A}{⋀}K_{i}K_{j}\sigma_{t}^{j}\underset{\left(j,k\right)\in E}{⋀}K_{i}K_{j}K_{k}\sigma_{t}^{k},\;\forall i\in A,$$ (14)

where *announce*(***x***) is an action symbolizing the announcement of all robots’ states. The common belief particles are updated from the announcement of all states to the multi-robot system such that

$$p_{ij,k}\leftarrow x_{k},\;\forall \left(i,j,k\right)\in A^{3}.$$ (15)

Similarly, all particles are updated according to the most recent public announcement, and the common belief set is updated so that

$$p_{ij,i}^{*}\leftarrow x_{i},\;\forall \left(i,j\right)\in E.$$ (16)

Since the common belief is updated to the world *w*_*t*_ shared according to (14), the particles in this set are propagated based on each robot’s status propositions. For example, in a two-robot team, if robot 1 communicates with robot 2 that it has found a task and will complete this task, robot 1 and robot 2 would propagate a common belief particle that moved to complete the task before continuing to cover the environment.

The local belief update is more complicated as all robots must also retain the common belief, $C_{i}$ , for partition consensus among disconnected robots. As such, the common belief is not updated upon receiving new information, but rather the second-order belief about each robot. Given that a robot has a belief about the current world, this belief is revised if an action changes robot *i*’s knowledge of the world

$$s_{t-1}^{i}⊗i\;:\;announce\left(x_{i}\right)\;\Rightarrow \;K_{i}\sigma_{t}^{i},$$ (17)

noting that knowledge and belief are equivalent $\left(B_{i}\sigma_{t}^{i}\equiv K_{i}\sigma_{t}^{i}\right)$ . In turn, a robot may communicate this action to only its connected neighbors

$$s_{t-1}^{i}⊗i\;:\;announce\left(x_{i}\right)=s_{t}⊧\underset{\left(i,j\right)\in E}{⋀}B_{i}B_{j}q_{i}^{\prime }\underset{\left(i,j\right)\in E}{⋀}B_{i}B_{j}B_{i}q_{i}^{\prime },$$ (18)

noticing that disconnected robots’ knowledge is not impacted, nor does robot *i* update its belief of the overall system and robot *j* updates its belief about robot *i* such that

$$p_{ji,i}\leftarrow x_{i}.$$ (19)

In this way, the system is able to maintain both local and global beliefs, even while disconnected using this announcement protocol. Also, the set $q_{ij,k}\in Q_{i}$ holds the timestamp that information was last shared between robot *i* and all other robots. Each particle in a connected team is assessed and revised if another robot has a more recent belief to ensure we can plan with the last shared belief. For example, if robot *j* finds a task and shares a new state with robot *i*, robot *i* will set all timestamps in the set *q*_*ij*,*j*_ to the current time and update its particle propagation for particle *p*_*ij*,*j*_ according to the new status of robot *j*, $\sigma_{t}^{j}$ , until assumed task completion. Then the particle will propagate toward its common belief, $c_{ij}\in C_{j}$ .

The maximum number of worlds in this epistemic model is the combination of all possible statuses in the system or $n\leq 4^{N_{r}}$ . Even this number is too large to track for a small multi-robot system, but, using dynamic epistemic logic, each *p*_*ij*,*k*_ is only updated upon an action in the action library, *A*.

**FIGURE 4:**
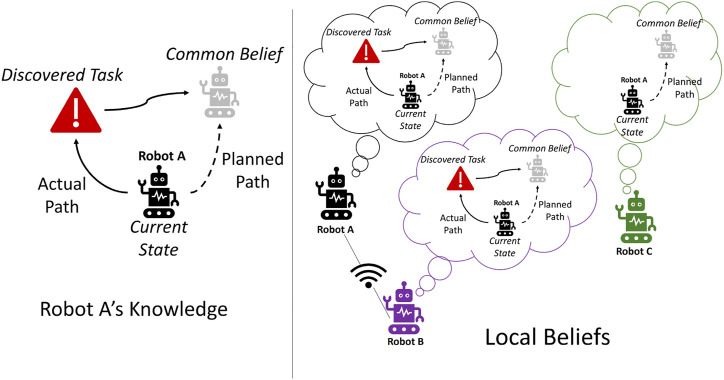
Illustration of a local belief update. Both robot A and robot B are connected and update their local shared belief, but retain the global common belief that achieves consensus with robot C.

Figure 4 illustrates the example of local belief update when a task is found and two robots are communicating while a third robot remains disconnected. For ease, in the figure, we display every robot’s belief about only robot A. Originally, robot A planned to follow the common belief, but upon discovering a task, it replanned to complete the task before continuing to track the common belief particle. Robot A and robot B are within communication range, and so robot A communicates that it will travel to complete the task before continuing to track the common belief. Robot B updates its belief about robot A (and *vice versa*). Robot C is not able to receive the updated information and continues to plan according to the common belief. Robots A and B propagate robot C’s belief, and robot A will eventually continue to track the common belief particle after completing the discovered task.

### 5.3.2 Epistemic planning

With our epistemic states and actions defined in Section 3.2 and the previous section, we now describe how these concepts can be used for planning. A planning task for robot *i* is defined by the tuple $\Pi =\left(s_{t}^{i},A,\gamma \right)$ , where *γ* is a goal formula. In plain language, the goal formula is completion of all tasks in the environment. The goal formulas are considered to be common knowledge, as each robot will act according to the same policies under the same conditions. Thus, we seek the following joint policy implementation, *π*, to ensure the completion of all tasks in the environment. The reason we use joint policies is that robots need to map indistinguishable epistemic states to the same actions. Therefore, we define the following rulesets.

First, robot *i* may discover a task requiring two robots and seek to communicate with robot *j* by traveling to its last shared belief. Consequently, $\sigma_{t}^{i}$ becomes *gossiping* and robot *i* travels to the particle with the most recent timestamp in the set $\left\{q_{ij,k}\forall k\in A\right\}$ . If robot *j* is not at its last shared belief, robot *i*’s belief about *j* is incorrect, and so additional worlds are possible and indistinguishable, given robot *i*’s current knowledge. Except for exhaustively searching for robot *j*, robot *i* does not have any way to find *j*. As a preface, we note that in order for this ruleset to be guaranteed to find an available robot, more robots than tasks need to be available because it may happen that the same subset of robots are simultaneously needed for different tasks. However, this ruleset will allow for effective operations if tasks are found asynchronously. Thus, we define the first ruleset as follows: if robot *j* is not found, *j* is excluded from its policy options *π* as robot *i*’s belief that robot *j* is available is false. Future planning excludes robot *j* as an option for completing the task since it must be operating according to another status such as gossiping to another robot or completing another task.

Second, robot *i* may discover a task, but believes robot *j* has also discovered the task first based on robot *j*’s coverage assignments. Therefore, robot *i* assumes the task has been accomplished by *j*. Upon communication, this assumption is verified and the task is designated for completion if it has not been accomplished. Thus, our acceptable common knowledge policy rulesets are established. The execution of *π* is defined as a maximal sequence satisfying the global formula *γ*. The algorithm for this sequence is defined in the following section.

### 5.4 Epistemic task allocation and gossiping

At the core of this framework is an epistemic-based multi-robot information dissemination and task allocation algorithm. As previously mentioned, in this paper, we focus on a subcategory of the MRTA problems known as the single-task multi-robot time-extended allocation problem. There are few mathematical models from combinatorial optimization research that tackle this further generalization of the assignment problem; however, the assignment problem can be modeled with joint, rather than per-robot, constraints for each task such that the utility, *u*(⋅), is maximized. The solution to the following assignment problem is the execution sequence of policy *π* satisfying the epistemic goal formula *γ* for completing all discovered tasks in the environment.

$$\;\;\max\underset{i\in A}{\sum }\underset{\tau \in T}{\sum }u_{i\tau }\left(t_{i\tau }\left(b_{i}\left(y_{i}\right)\right)\right)y_{i\tau },$$ (20)

$$\begin{array}{ll} \;\text{s.t.} & \;\underset{i\in A}{\sum }\underset{\tau \in T}{\sum }y_{i\tau }\geq N_{\tau },\;\;\forall \tau \in T\\ & \;t_{i\tau }\left(b_{i}\left(y_{i}\right)\right)\geq t_{i\varsigma }\left(b_{i}\left(y_{i}\right)\right)+\delta_{\varsigma \tau }\;\forall \left(\varsigma ,\tau \right)\in S_{i}\\ & \;t_{i\tau }\left(b_{i}\left(y_{i}\right)\right)\geq 0\;\forall \tau \in T\\ & \;x_{i\tau }\in \left\{0,1\right\}, \end{array}$$ (21)

where *y*_*iτ*_ = 1 if robot *i* is assigned to task *τ* and $y_{i}=\left\{y_{i1},\ldots ,y_{iN_{t}}\right\}$ . The arrival time for the *i* ^th^ robot is a unique function, *t*_*iτ*_, that accounts for the arrival time of *N*_*τ*_ necessary robots for task *τ*. The variable *δ*_*ςτ*_ is the duration between tasks *ς* and *τ*. The order of tasks is represented by a directed graph, *S*_*i*_, created by the order of robot *i*’s path, ***b***_*i*_, where an edge in *S*_*i*_ is (*ς*, *τ*), which indicates that task *ς* is performed before task *τ*.

Additionally, when a task is discovered, a robot must consider if any assistance is required to complete a task, any tasks that are already in its queue, and prior communicated allocations of tasks to other robots. If assistance is required, the robot must disseminate the new information to neighboring robots, acting as an *ad hoc* network by visiting a neighboring robot’s belief state.

To account for these considerations, the following section describes each of the three steps involved in our proposed algorithm: i) initial task bundling to assign each task to a robot, ii) makespan minimization to minimize the expected time to complete all tasks, and iii) a gossip protocol algorithm to optimize the assignment of information dissemination.

#### 5.4.1 Task bundling

First, we require a valid initial solution for the task allocation problem. We define a robot’s bundle as an ordered list of tasks to complete. Given that each task may require more than one robot, the allocation order requires that one task must be executed in the bundle order before another is assigned. Thus, to accommodate this temporal constraint, we use a modified sequential-single item (SSI) auction for initial bundling as shown in Algorithm 1. The task bundling algorithm initializes an empty bundle for each robot, and each robot bids on the first task in the set of locally discovered tasks, D⊆T . The highest *N*_*τ*_ bidders incorporate the task at the end of their bundle (lines 6–10).

Algorithm 1 Initial Task Bundling Algorithm

**Require:** *N*_*τ*_ ⊳ number required for task τ∈D

1: $B_{j}=\emptyset ,\forall j\in A$ ⊳ initial bundle
2:  **for each** τ∈D **do**
3:   **for each** j∈A **do**
4:    Bid on task with utility *h*(*x*_*j*_, *τ*)
5:   **end for**
6: $W_{\tau }=\left\{j\in A:\;\;|\left\{j^{\prime }\in A:h\left(x_{j^{\prime }}\right)<h\left(x_{j}\right)\right\}|\leq N_{\tau }\right\}$

7:   **for each** j∈A **do**
8:    **if** *j* ∈ *W*_*τ*_ **then**
9:     *B*_*j*_ ← *B*_*j*_ *⊕*_*end*_ *τ*
10:    **end if**
11:   **end for**
12:  **end for**

If a robot is not connected to make a bid, the locally connected team member with the highest confidence (i.e., most recent information documented by the set Q ) of the state of the disconnected robot submits a bid on their behalf. The bid for adding task *τ* to robot *j* is defined by marginal improvement of robot *j*’s bundle score. As such, the bid is defined as

$$h\left(x_{j},\tau \right)=\lambda_{\tau }-S_{j}^{B_{j}{⊕}_{\mathrm{end}}\left\{\tau \right\}},$$ (22)

where $S_{j}^{B_{j}}$ is initialized to $S_{j}^{\emptyset }=0$ and denotes the cost of traveling, given the original bundle, *B*_*j*_, and the added task. The operator *⊕*_*end*_ adds the antecedent task *τ* to the end of its precedent bundle, *B*_*j*_. This decentralized algorithm allows connected robots to quickly create a valid task allocation, but does not account for the completion time of every task. Thus, minimizing the makespan of the bundle order will reduce the task allocation’s estimated completion time.

#### 5.4.2 Makespan minimization

Makespan is the time taken for all robots to finish all of their assigned tasks (Nunes and Gini, 2015). Attempting to minimize the makespan of the bundled tasks accounts for a scenario where a robot can complete a task “on the way” to another task and complete all assigned tasks faster. Algorithm 2 gives an overview of the makespan minimization algorithm.

Algorithm 2 Makespan minimization

**Require:** $B_{j}\;\forall j\in A$ ; *m*_*best*_ = *makespan*(*B*_*j*_)
1:  **for each** j∈A **do**
2:   *B*_*tmp*_ ← *B*_*j*_
3:   **for each** *j*′ ∈ *B*_*j*_ **do**
4:    *B*_*tmp*_ ← *B*_*j*_ \ *j*′
5:    **for each** *n* ∈ len(*B*_*j*_) **do**
6:     *B*_*tmp*_ ← *B*_*tmp*_ *⊕*_*n*_ *j*′
7:     *m*_*tmp*_ = *makespan*(*B*_*tmp*_)
8:     **if** *m*_*tmp*_ < *m*_*best*_ **then**
9:      *B*_*j*_ ← *B*_*tmp*_
10:      *m*_*best*_ ← *m*_*tmp*_
11:     **end if**
12:    **end for**
13:   **end for**
14:  **end for**

The algorithm iterates through all tasks in each robot’s bundle and places each task in each available path segment. Then, the makespan is calculated for the robot’s new bundle order, *B*_*tmp*_. If the new makespan, *m*_*tmp*_, is smaller than the best makespan, *m*_*best*_, the bundle *B*_*j*_ is replaced with *B*_*tmp*_. It should be noted that the order of tasks that were previously communicated to now disconnected robots must be maintained in Algorithm 2 by not reordering these tasks in the makespan minimization (lines 3–13). To maintain this order, valid *j*′s are tasks that have not been previously assigned and ordered to disconnected entities (i.e., valid tasks have yet to be gossiped to allocated robots).

The ordered bundle for each robot would typically be the execution sequence for policy *π* to complete the NP-hard problem defined in 20, but given the communication restriction, communication assignments must also be allocated for every robot to perform its sequence of tasks. For this reason, we introduce the gossip protocol assignment algorithm.

#### 5.4.3 Gossip protocol

If a robot is assigned to a task, but is not aware of the new information, robots in charge of the allocation must deliver the information, acting as an *ad hoc* network and informing the necessary team of robots through a gossip protocol-based algorithm, accounting for the cascading effect of communication and adding nodes to the *ad hoc* network. Algorithm 3 steps through the allocation of peer-to-peer communication tasks based on the resulting task allocation from Algorithm 2. Similar to bids in Algorithm 1, the robots with the most recent state information for a disconnected robot will submit bids on their behalf.

Algorithm 3 Gossip protocol auction

**Require:** robots *j*_*g*_ ∈ {*G*_*j*_: *B*_*j*_ ≠ ∅}
1:  *D* = *R*_*c*_, where *R*_*c*_ are the connected robots
2: $G_{B_{j}}=\emptyset$ is the gossiping assignments for robot j given bundle *B*_*j*_
3:  **while** *D* ≠ *G*_*j*_ **do**
4:   **for each** *j*_*g*_∉*D* **do**
5:    **for each** *j*_*v*_ ∈ *D* **do**
6:     *b*_*vg*_ = *bid*(*j*_*v*_, *j*_*g*_)
7:    **end for**
8:   **end for**
9:   **for each** *j*_*v*_ ∈ *D* **do**
10:    g* = argmax_*g*_(*b*_*vg*_)
11:    **if** $j_{g^{*}}\notin D$ **then**
12: $G_{B_{j_{v}}}=G_{B_{j_{v}}}{⊕}_{\mathrm{end}}j_{g^{*}}$

13:     *D* ← *D⊕*_*end*_ *j*_*g*_
14:    **end if**
15:   **end for**
16:  **end while**

First, the set *G*_*j*_ is defined as the robots who are assigned a task in the bundle. The variable *D* represents the set of robots who either know the information to be disseminated or a robot has been assigned to communicate with them. The set $G_{B_{j}}$ is initialized as the currently connected robots in *G*_*j*_, and a new empty gossip bundle is established for all robots (lines 1–2). Next, each required robot, *j*_*g*_, is bid on by a robot, *j*_*v*_, in the *D* set (lines 4–8). The highest bid for robot *j*_*g*_∉*D* is added to robot *j*_*v*_’s bundle, and *j*_*g*_ is added to the *D* set (lines 9–15). The while loop repeats until all necessary robots for *B*_*j*_ have been assigned and accounts for the cascading effects of communication (i.e., when a robot has communicated with another robot, two robots are now available to gossip to other members).

After execution of these algorithms, the execution policy for a robot *i* is represented as a sequence that is defined by the concatenation of its gossip bundle $G_{B_{i}}$ and task bundle *B*_*i*_. A robot is responsible for its communication assignments before continuing to its ordered task execution. The ordered sequence for every robot is the execution of *π* satisfying the goal formula *γ*, given its current epistemic state $s_{t}^{i}$ .

Task allocation, makespan minimization, and coverage of an environment are all NP-hard problems. As such, we reflect on the size of the solution space. Given a set of discovered tasks $T_{D}$ with each *τ* task requiring *N*_*τ*_ robots, the system needs a maximum number of ∑τ∈TDNaNτ to find the best solution to the allocation problem. The problem increases in complexity as we include the gossip protocol, which requires robots to communicate tasks to allocated robots. In total, the number of solutions for any number of tasks and robots with gossip protocol is (*N*_*a*_(*N*_*a*_ + *N*_*t*_))!. The output of the proposed algorithms is a heuristic to find a feasible solution. However, we note that as the number of robots and tasks increases, the solution speed decreases and that a full recompute is required if new information is made available to the robots.

## 6 Simulations

In this section, we provide comparisons from MATLAB simulations with our approach implemented on two case studies. Case Study I is a simulated scenario where all robots know that only one task exists in the partially known environment requiring an unknown number of robots at an unknown location. Case Study II is a simulated scenario similar to Case Study I, but there are an unknown number of tasks that, in total, do not require more robots than are available, $\sum_{\tau \in N_{t}}N_{\tau }\leq N_{a}$ . Simulations were performed on 15 randomly generated cluttered 50 m × 50 m environments with 10–20 1 m × 1 m initially unknown obstacles. For repeatability, we set *η*_*g*_ at 1 and *η*_*o*_ at 5 for the APF behavior introduced in (6) and (7).

The proposed approach is compared against two other methods. The first method applies a constant connectivity constraint, not allowing agents to travel outside of a 10 m communication range. The second method assumes ideal conditions, where robots can communicate across the entire environment. In the following section, we refer to the first method as the “flock” method and the second method as the “ideal” method. Both methods use smooth A* path planning and an artificial potential field technique for controlling the robots toward uncovered regions and away from obstacles. In all methods, the maximum velocity is 3 m/s, simulated Lidar range is 5 m, and the robots’ motion is modeled with a single-integrator model with ***ν*** ∼ *N*(0, 0.2) from (1). All videos of the simulations presented in this section are available in Supplementary Material S1 and on our website^1^.

### 6.1 Case study I: multi-robot single task

**FIGURE 5:**
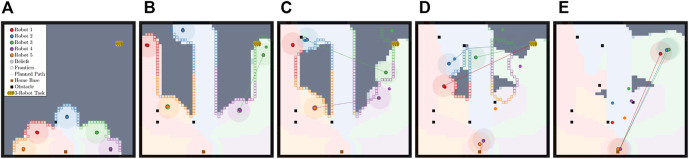
This figure depicts the progression of the Case Study I simulation where there is a single task in the environment. **(A)** shows the starting state of the robots after initial disconnection. **(B)** shows robot 3 finding the 3-robot task and deciding to communicate with robot 4. **(C)** shows successful communication and replanning with robot 4 tasked with communicating to robot 5 and robot 3 to robot 2. **(D)** shows all robots with their final assignment with robots 1, 2, and 3 assigned to the task, while robots 4 and 5 route to home base. **(E)** shows that with the task completed, all robots are routed home.

In Case Study I, all robots are aware that only one task is located in the environment. This simulated scenario is similar to a search and rescue mission, where the goal is to locate and rescue an individual at an unknown location in a large environment. The robots begin by covering the area and, upon discovery, calculate how many robots are necessary for the rescue operation. Then, the robot disseminates the information to the rest of the robot team, who either are tasked with returning to home base or assisting in the rescue. An example scenario is shown in Figure 5. The example showcases the proposed method when only one task is in the environment. The robots begin by disconnecting to more efficiently cover the environment. Robot 3 finds a task, as shown in Figure 5B, and plans using its knowledge of each robot’s epistemic state. The result is for robot 3 to communicate with robot 4 via robot 4’s belief state. After communication is successful, as shown in Figure 5C, robots 3 and 4 similarly plan to communicate with robots 2 and 5 via their respective belief states. Lastly, all robots are assigned to the final task shown in Figure 5D and complete the task shown in Figure 5E before routing to home base.

**FIGURE 6:**
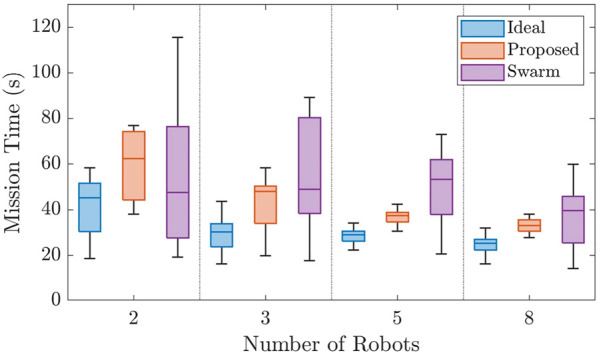
Figure comparing the results of the simulated scenarios for Case Study I. The proposed framework is measured against two other methods and shown to decrease the variance over random environments and decrease mission time as the robot team grows in size when compared to the always connected flock.

This scenario was implemented using two, three, five, and eight robot teams in 15 varying environments. The results of the simulated method comparisons are shown in Figure 6.

The figure illustrates the proposed framework’s performance, given a variety of environments and team sizes. The proposed method decreases the variance in the mission time with a two-robot team, but is outperformed by the flock method since the robots remain together and can become lucky, finding the task and completing the mission. This method even outperforms our ideal scenario in some cases since the robots must potentially travel a longer distance to the task once found by a team member. However, as the robot teams become larger, the flock method is outclassed by more efficient coverage of the environment, represented by the ideal and proposed case. We also notice that the variance in mission times of the proposed and ideal methods is similar with a standard deviation of 11 s and 14 s, respectively, across all robot team sizes. In comparison, the standard deviation of the flock method is 48 s. Additionally, though initially outperformed with a team size of two robots, the proposed method on average outperforms the flock method by 13 s. The ideal method also is 11 s faster on average than the proposed method.

### 6.2 Case study II: multi-robot multi task

**FIGURE 7:**
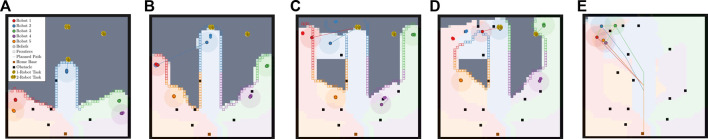
This figure illustrates the progression of the Case Study II simulation where the number of tasks is unknown. **(A)** shows the starting state of the robots after initial disconnection. **(B)** shows robot 1 finding the 2-robot task and deciding to communicate with robot 2. **(C)** shows successful communication, and **(D)** shows task completion and the robots route to their home base **(E)**.

In Case Study II, all robots do not know how many tasks are in the environment, where the tasks are located, or how many robots are required at each task. This simulated scenario is a recovery of an asset that may be scattered across a large environment. The robots begin by covering the area and, upon discovery of a task, calculate how many robots are necessary for the rescue operation. Then, the robot disseminates the information to the necessary members of the robot team. If a robot is not able to be located at its believed location, the robot considers this robot occupied and does not consider it in the next iteration of assignments. The robots must cover the entire environment in order to identify if any tasks lie in the uncovered portions of the environment. Figure 7 presents an example scenario from the comparison scenarios. Figure 7 exhibits a scenario with three tasks at unknown locations. Two tasks require one robot and one task requires two robots. The individual tasks are completed upon discovery by the closest robot. When robot 2 discovers the two-robot task in Figure 7B, it routes to robot 1’s belief state to ask for assistance. Both robots travel to complete the task shown in Figure 7C, and robot 3 finds the last single robot task in the environment shown in Figure 7D. After all portions of the environment have been covered, all robots route to their home base.

**FIGURE 8:**
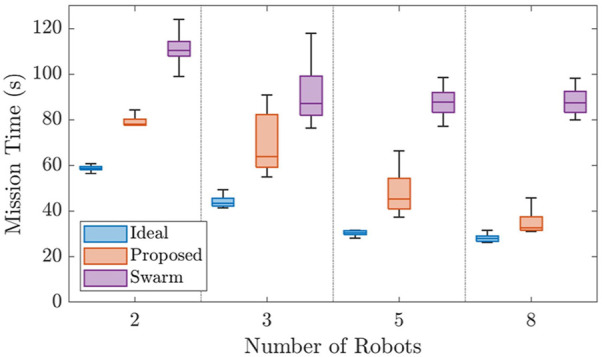
Figure comparing the results of the simulated scenarios for Case Study II. The proposed framework is shown to decrease mission time drastically when compared to the always connected flock and perform similarly to the ideal method as team size increased.

Case Study II was also implemented using two, three, five, and eight robot teams in 15 varying environments. The results are shown in Figure 8.

This Figure displays a strong argument for the proposed method when compared to the flock and ideal methods. On average, the proposed method performed only 16 s slower than the ideal method, even considering the communication limitation. Additionally, the proposed method was 35 s faster than the flock method. Though the variance for the proposed method was higher than that of the flock and ideal methods, its worst case mission time is approximately as good as the median performance of the flock method.

## 7 Experiments

The proposed approach was also validated through laboratory experiments with a multi-robot team. The team consists of two to three Husarion ROSbot 2.0 UGVs using a Vicon motion capture system. The experiments effectively demonstrate all parts of the proposed approach, including intentional disconnections, searching, and gossiping behaviors. In all experiments, the UGVs start within communication range and are tasked to cover the environment and complete any discovered tasks. Experiments were performed in a 4 m × 5.5 m space containing convex obstacles considering, as a proof of concept, a sensing and communication range for each robot of 1 m. All videos of the experiments presented in this section and more are available in the provided Supplementary Material S1 and on our website^2^.

### 7.1 Case study I: multi-robot single task

**FIGURE 9:**
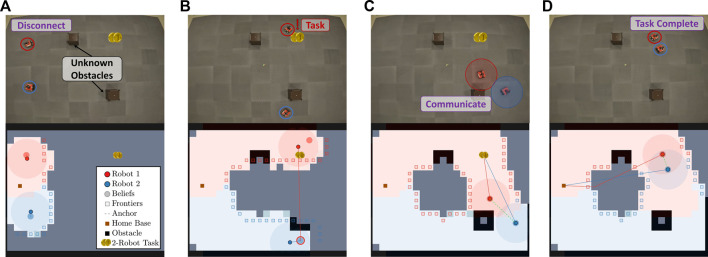
This figure illustrates the progression of the Case Study I experiment where there is only one task. **(A)** shows the starting state of the robots after initial disconnection. **(B)** shows robot 1 finding the 2-robot task and deciding to communicate with robot 2. **(C)** shows successful communication, and **(D)** shows task completion and the robots route to their home base.

Displayed in Figure 9 are the results from an experiment with a two-robot team. The columns of Figure 9 correspond to different instances within the experiment, and each row from the top to bottom shows snapshots of the robots at different times throughout the experiment and the current map of the environment covered by the team.

As shown in the figure, the robot team initially disconnects to more efficiently cover the environment. As shown in Figure 9B, robot 1 finds a task and plans to gossip the new information to robot 2. Figure 9C shows the robots communicating and traveling to complete the task. Lastly, as shown in Figure 9D, the robots complete the task and return to their home base.

**FIGURE 10:**
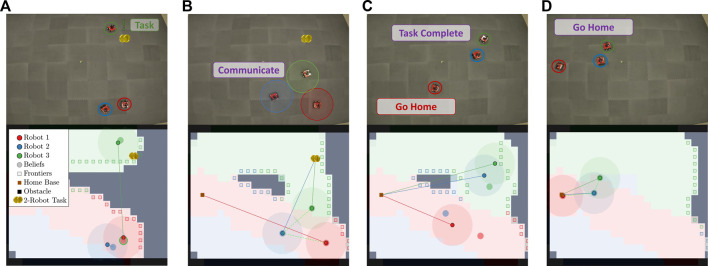
This figure illustrates the progression of the Case Study I experiment with a three-robot team and one task. **(A)** shows robot 3 finding a 2-robot task. **(B)** shows robot 3 communicating to robot 1 and 2 and allocating robot 2 and 3 to the task while sending robot 1 home. **(C)** shows successful task completion, and in **(D)** all the robots return to their home base.

Additionally, we show an experiment with a three-robot team. Unknown obstacles were not included in three-robot experiments due to limited space, but the method remained the same for the entire duration of the experiment. The columns of Figure 10 correspond to different instances within the experiment, and each row from the top to bottom shows snapshots of the robots at different times throughout the experiment and the current map of the environment covered by the team. As shown in the figure, the robot team initially disconnects to more efficiently cover the environment. As shown in Figure 10A, robot 3 finds a task and plans to gossip the new information to robot 1. Figure 10B shows the robots communicating and allocating robots 2 and 3 to complete the task while robot 1 returns home. As shown in Figure 10C, the task is completed, and, as shown in Figure 10D, all robots return to their home base.

### 7.2 Case study II: multi-robot multi task

**FIGURE 11:**
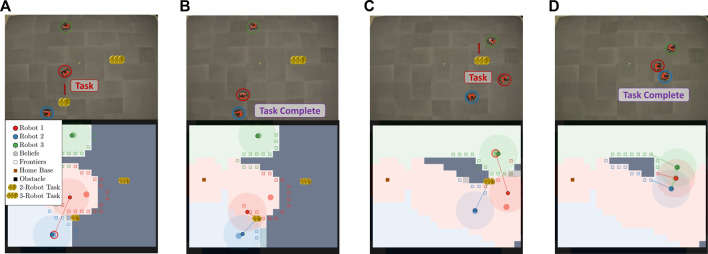
This figure illustrates the progression of the Case Study II experiment where the number of tasks is not known. **(A)** shows robot 1 finding a 2-robot task after initial disconnection and planning to communicate with robot 2. **(B)** shows robot 1 and robot 2 completing the task and planning to return to their belief states. **(C)** shows robot 1 and robot 2 finding a 3-robot task while connected and planning for robot 1 to communicate with robot 3. **(D)** shows the 3-robot task completed. Subsequently, all agents finish coverage of the environment and return to the home base.

We also show an example experiment with a three-robot team in Figure 11. Both the locations of the obstacles and the number of tasks to complete are unknown here. Therefore, the robots are tasked with covering the environment, gossiping to necessary team members, and completing discovered tasks. The environment has two tasks, one that requires two robots and one that requires three robots to complete.

In this example experiment, robots intentionally disconnect to cover the environment more efficiently. After a short time, robot 1 finds a task that requires two robots. Robot 1 plans to tell its believed closest neighbor and gossips to robot 2, as shown in Figure 11A. Figure 11B shows that the robots who know about the task complete it and travel back to their global belief states. Figure 11C shows robot 1 finding a three-robot task while connected to robot 2. Robot 2 is allocated immediately to the task, and robot 1 is tasked with gossiping the new discovery to robot 3. All robots converge to complete the task and finish covering the environment before returning to home base, shown in Figure 11D.

## 8 Conclusion and future work

In this work, we have presented a novel framework for multi-robot systems that leverage epistemic planning to allow for each robot to incorporate depth-of-reasoning in its mission planning framework. The proposed method allows a multi-robot system to disconnect and cooperatively plan according to a set of belief and empathy states. The beliefs are propagated using a Frontier-based method for coverage of a partially unknown environment and updated using dynamic epistemic logic and planning. The dynamic epistemic task allocation algorithm utilizes these belief states in allocating tasks discovered in the environment to satisfy the epistemic planning task. This enables dynamic task allocation to be performed while disconnected. A robot subsequently plans to communicate the allocation by traveling to the belief state.

In the simulations and experiments, we show the validity and applicability of our approach when compared with perfect communication and a standard flock method, where a robot must not travel outside of the communication of the multi-robot system. Our results, given an unknown number of tasks in the environment, show a drastic decrease in mission time when compared to the flock method and comparable results to scenarios where communication is always available. The results for the single-task case study also showed improvement in overall mission time, but greatly decreased to variance in mission time from the swarm method. Hence, thanks to the proposed epistemic planning framework, it is possible to act closely to the ideal case of always connected systems while letting each robot explore the environment more efficiently.

From here, future theoretical work includes addressing the challenges of dynamic task lengths and improving strategies for additional considerations such as failures, disturbances, or fully unknown environments. We also plan to decrease the computation time for task allocation and optimize the necessary belief propagation for a larger multi-robot system by dividing the team into sub-teams. Further modeling of epistemic planning using epistemic Markov decision processes to reach consensus, given probabilistic communication models, failures, and complex unknown obstacles, is also in our agenda.

## Data Availability

The original contributions presented in the study are included in the article/Supplementary Material, further inquiries can be directed to the corresponding author.

## Footnotes

1
https://www.bezzorobotics.com/lb-frontiers23.

2
https://www.bezzorobotics.com/lb-frontiers23.
