# Supplementary figures and images for: Epistemic planning for multi-robot systems in communication-restricted environments
Source: Front Robot AI. 2023 May 23;10:1149439. doi: 10.3389/frobt.2023.1149439 (PMC10243298; doi:10.3389/frobt.2023.1149439)

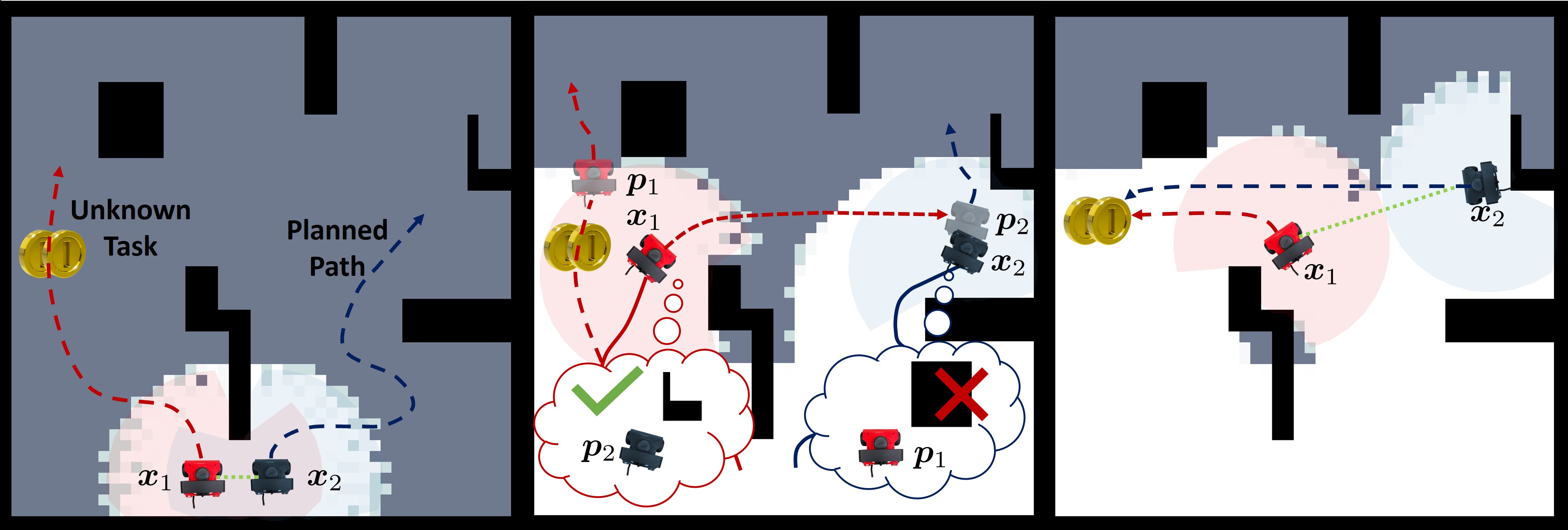

Supplement: Supplementary file 1 [file DataSheet1.ZIP › OrderedPictures/0IntroPicAttempt2.jpg]

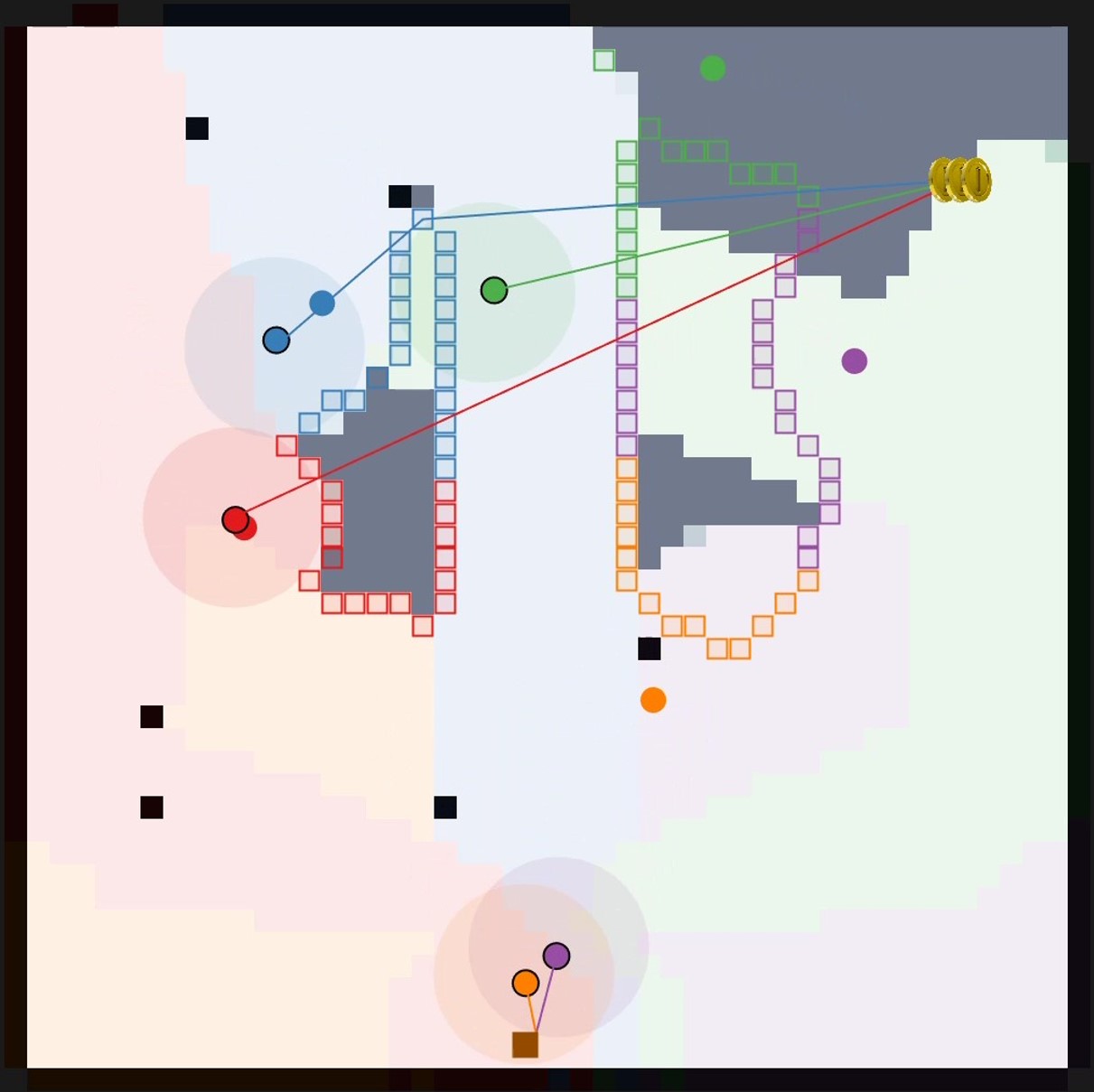

Supplement: Supplementary file 1 [file DataSheet1.ZIP › OrderedPictures/10MASTSimExamp3Final.jpg]

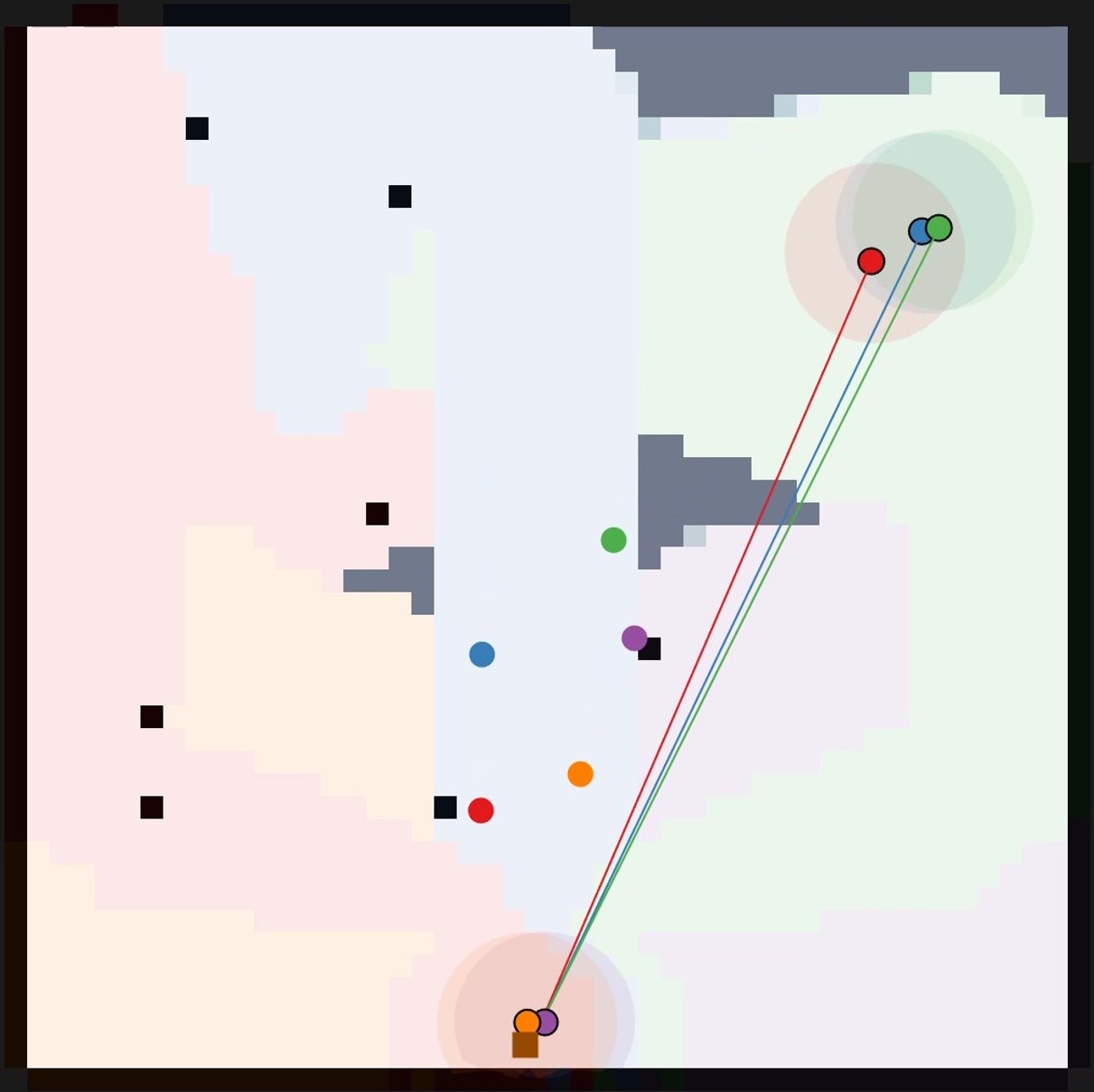

Supplement: Supplementary file 1 [file DataSheet1.ZIP › OrderedPictures/11MASTSimExamp4Final.jpg]

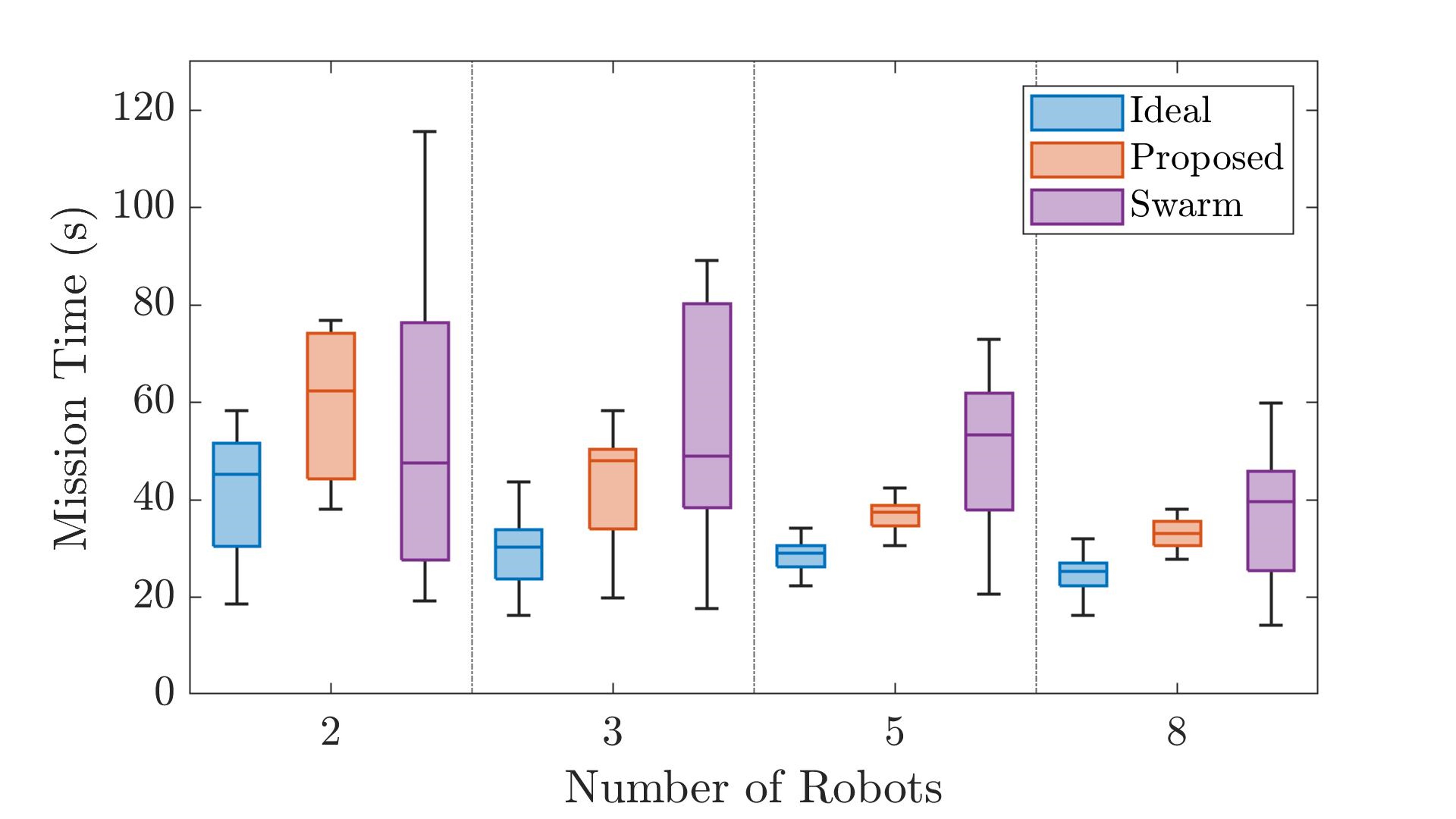

Supplement: Supplementary file 1 [file DataSheet1.ZIP › OrderedPictures/12MAST_BoxPlotFinal.jpg]

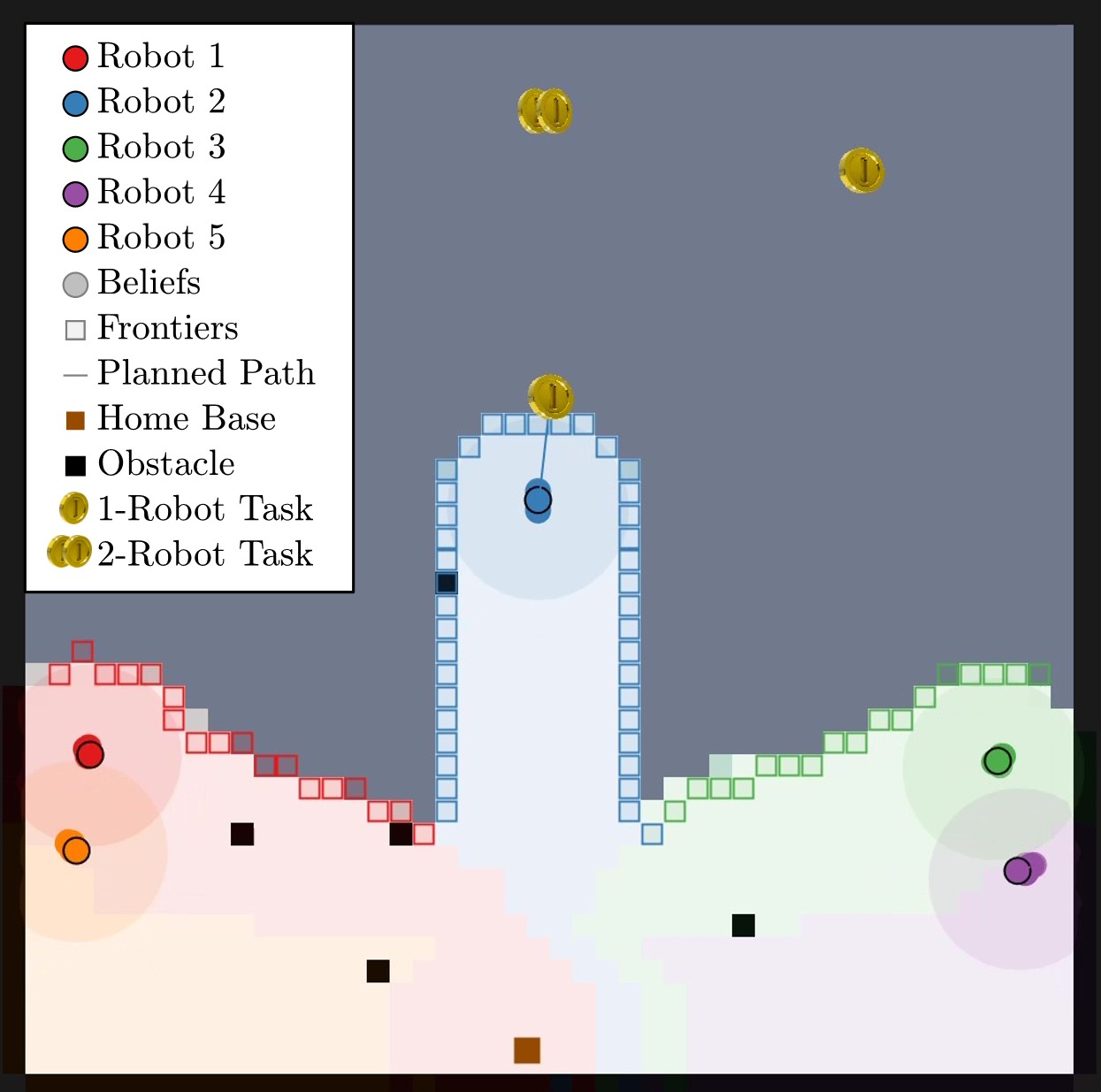

Supplement: Supplementary file 1 [file DataSheet1.ZIP › OrderedPictures/13MAMTSimExamp1_Final.jpg]

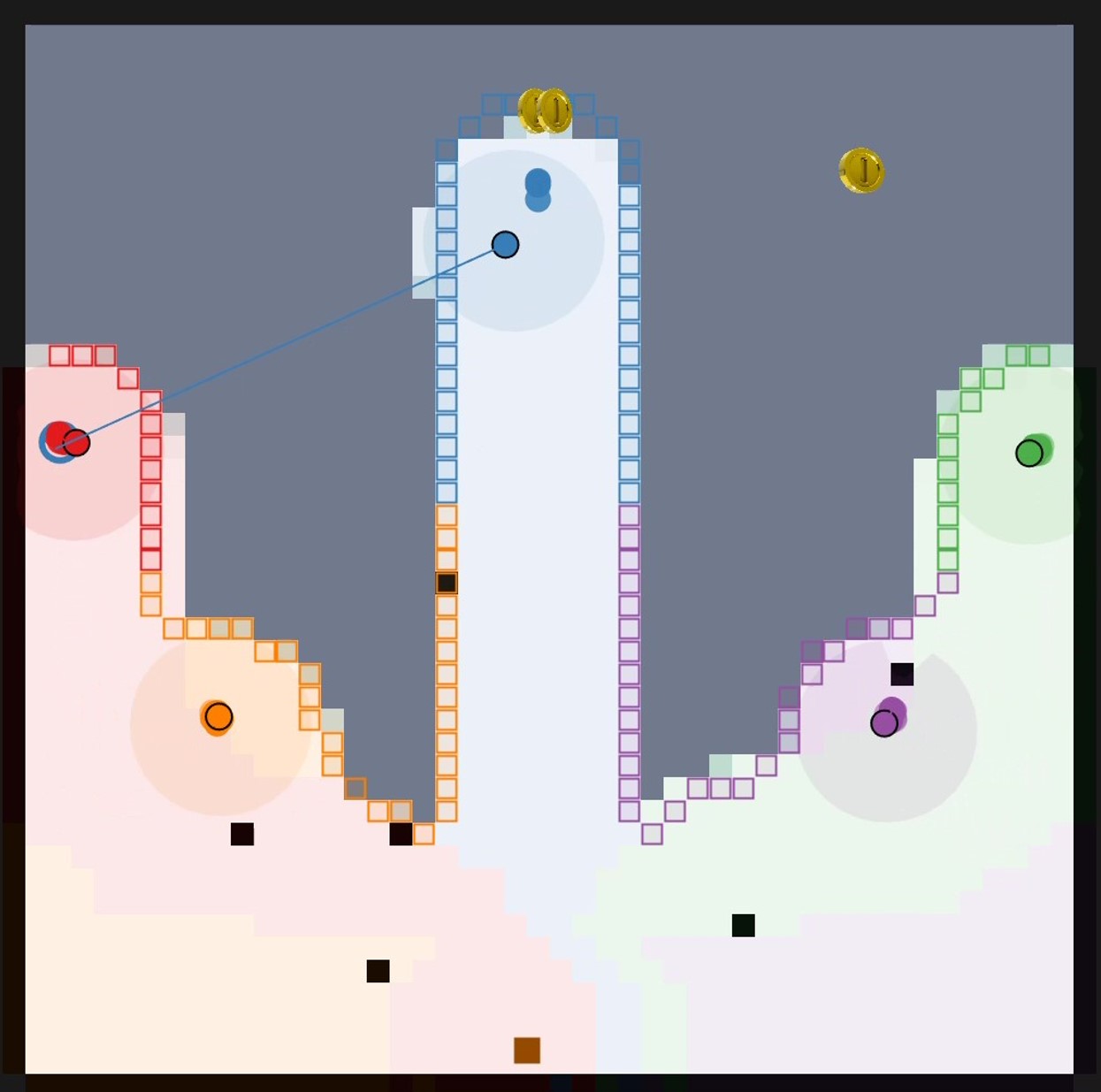

Supplement: Supplementary file 1 [file DataSheet1.ZIP › OrderedPictures/14MAMTSimExamp2_Final.jpg]

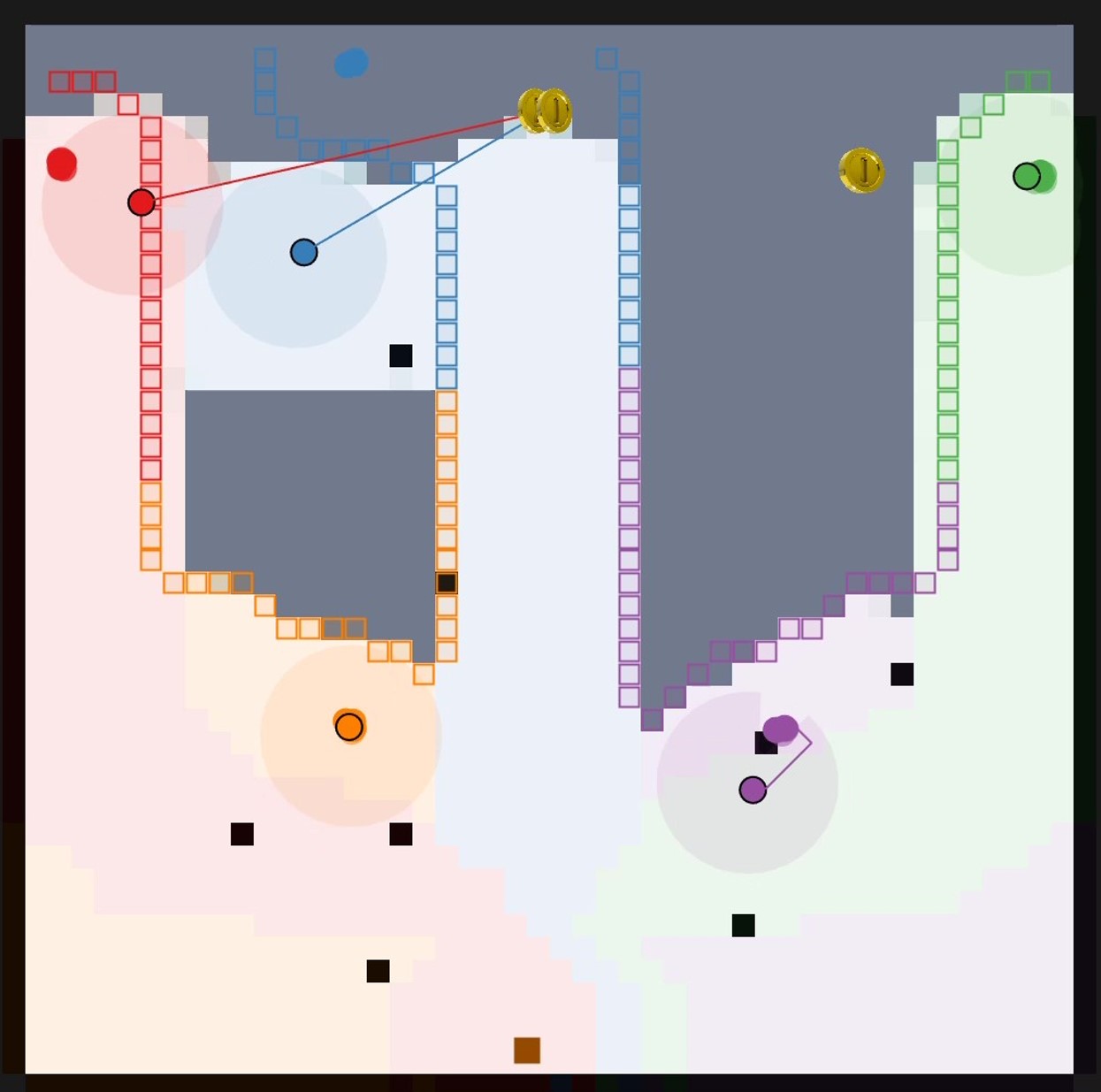

Supplement: Supplementary file 1 [file DataSheet1.ZIP › OrderedPictures/15MAMTSimExamp3_Final.jpg]

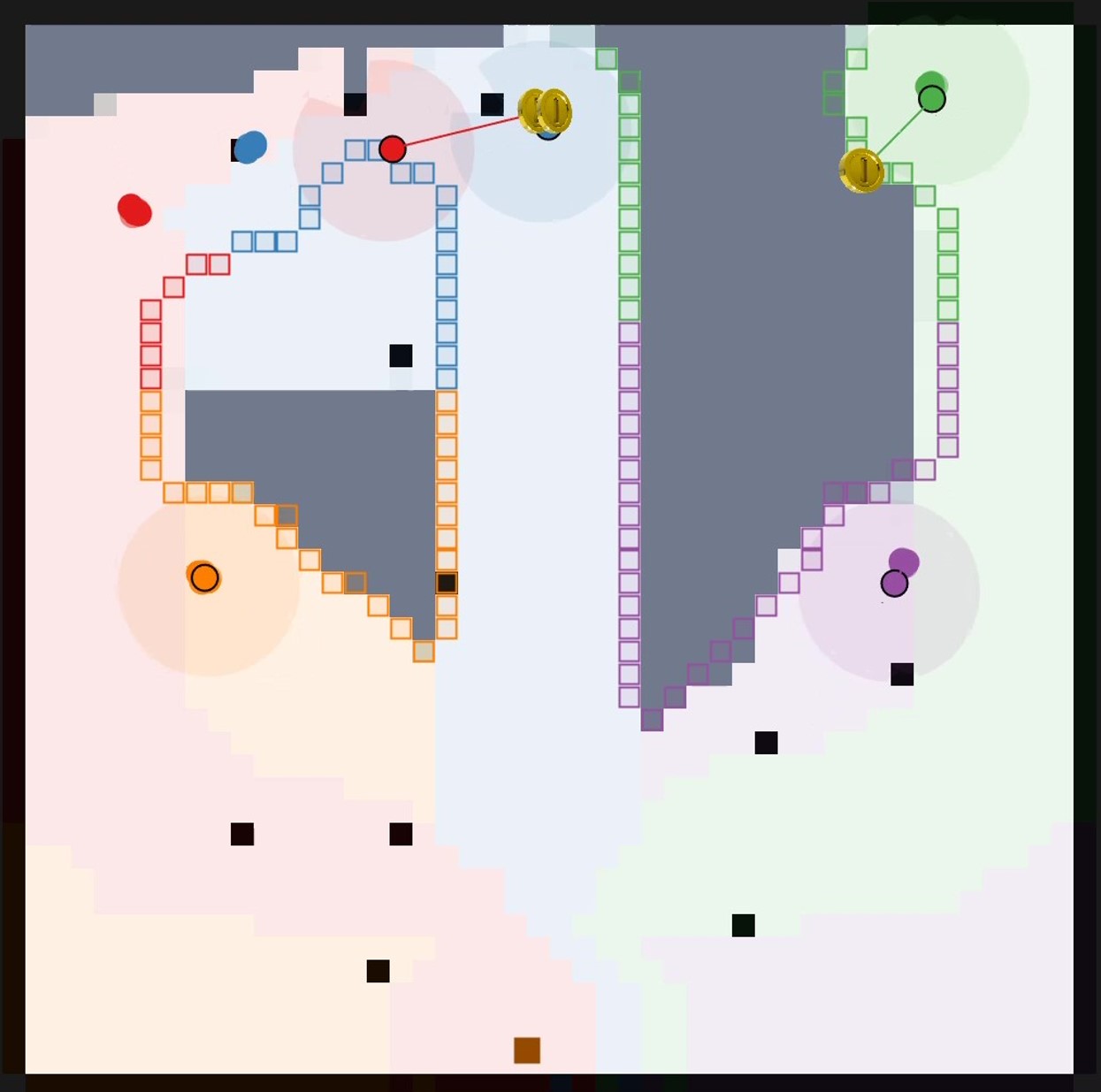

Supplement: Supplementary file 1 [file DataSheet1.ZIP › OrderedPictures/16MAMTSimExamp4_Final.jpg]

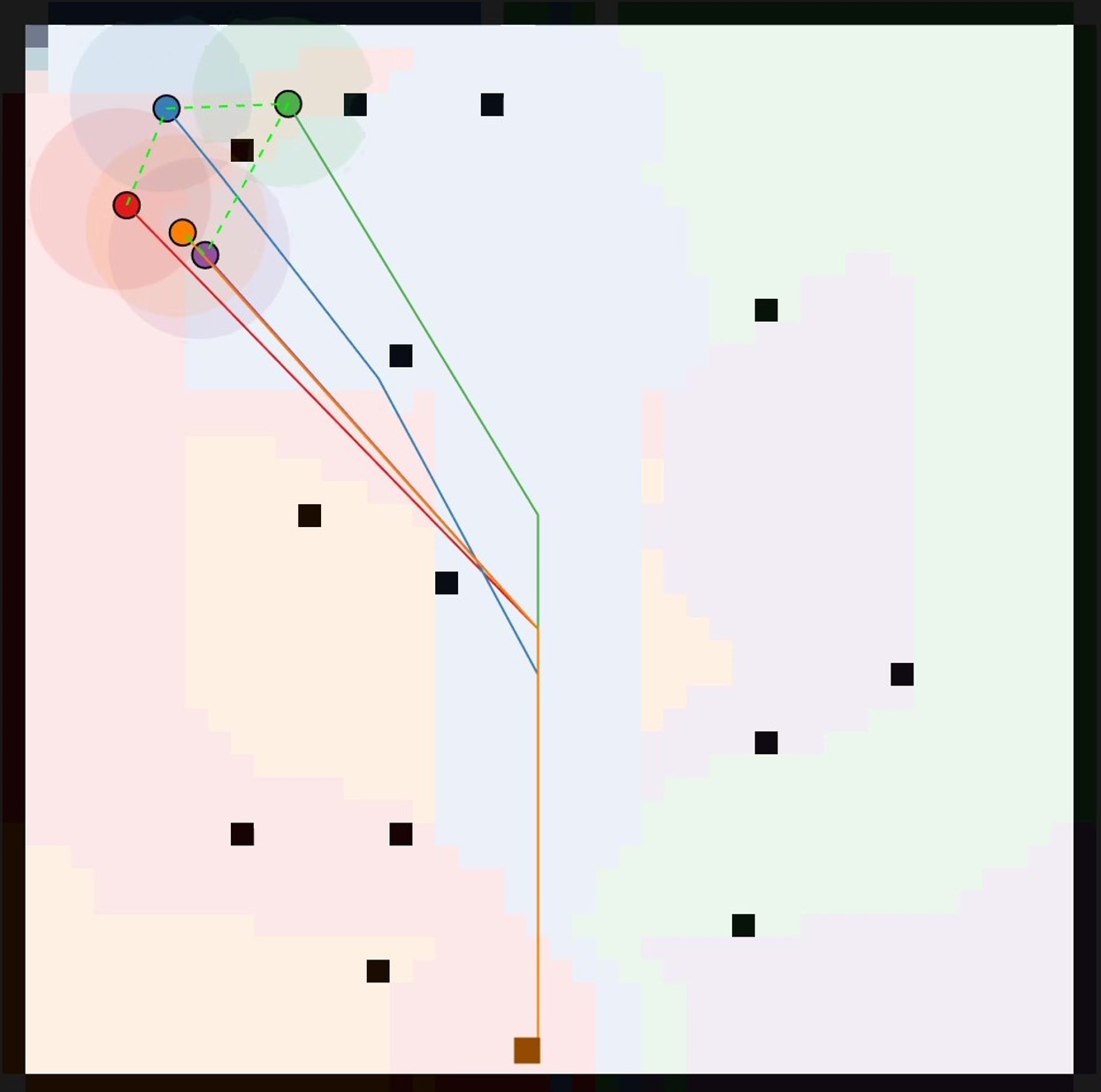

Supplement: Supplementary file 1 [file DataSheet1.ZIP › OrderedPictures/17MAMTSimExamp5_Final.jpg]

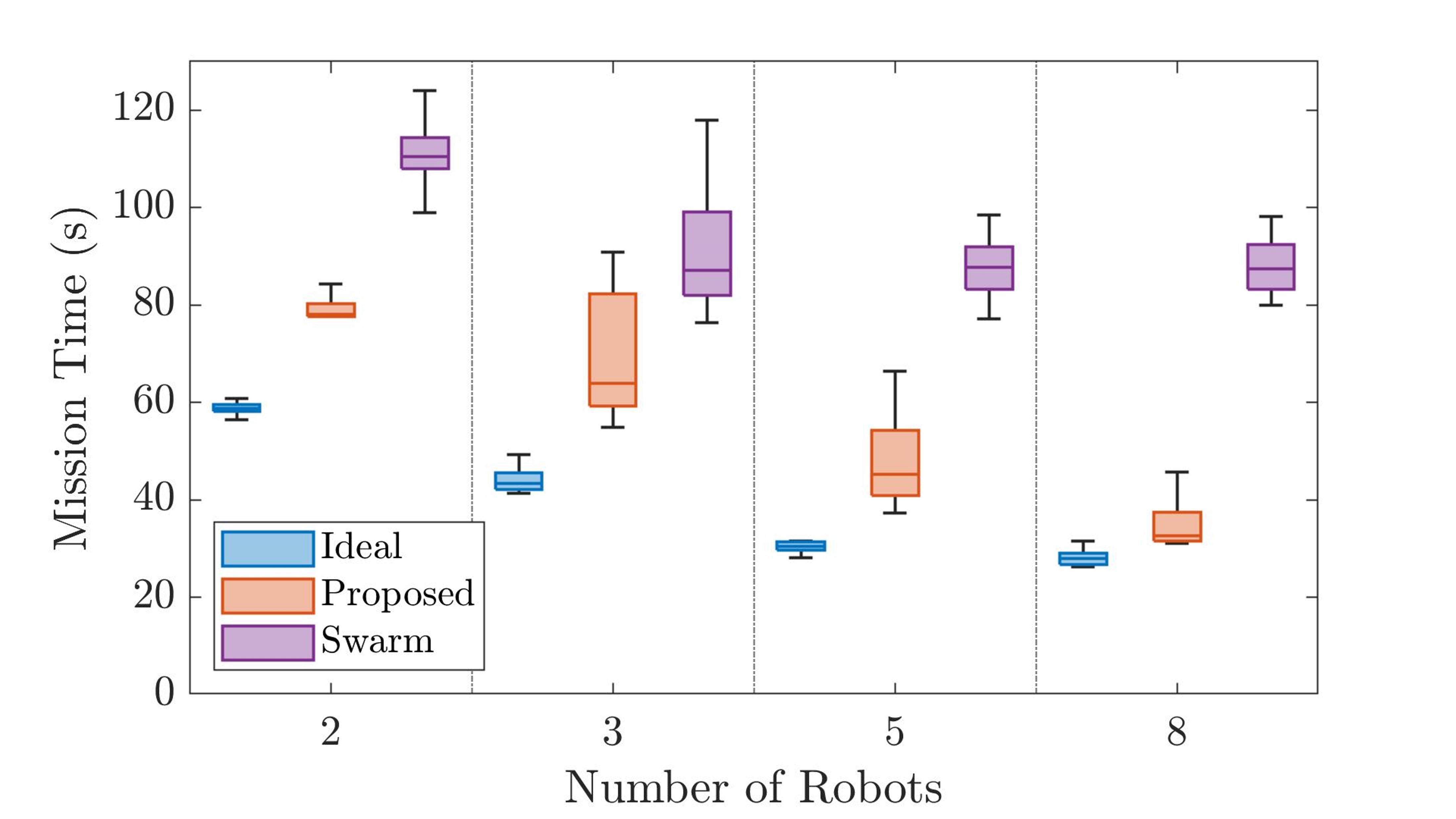

Supplement: Supplementary file 1 [file DataSheet1.ZIP › OrderedPictures/18MAMT_BoxPlotFinal.jpg]

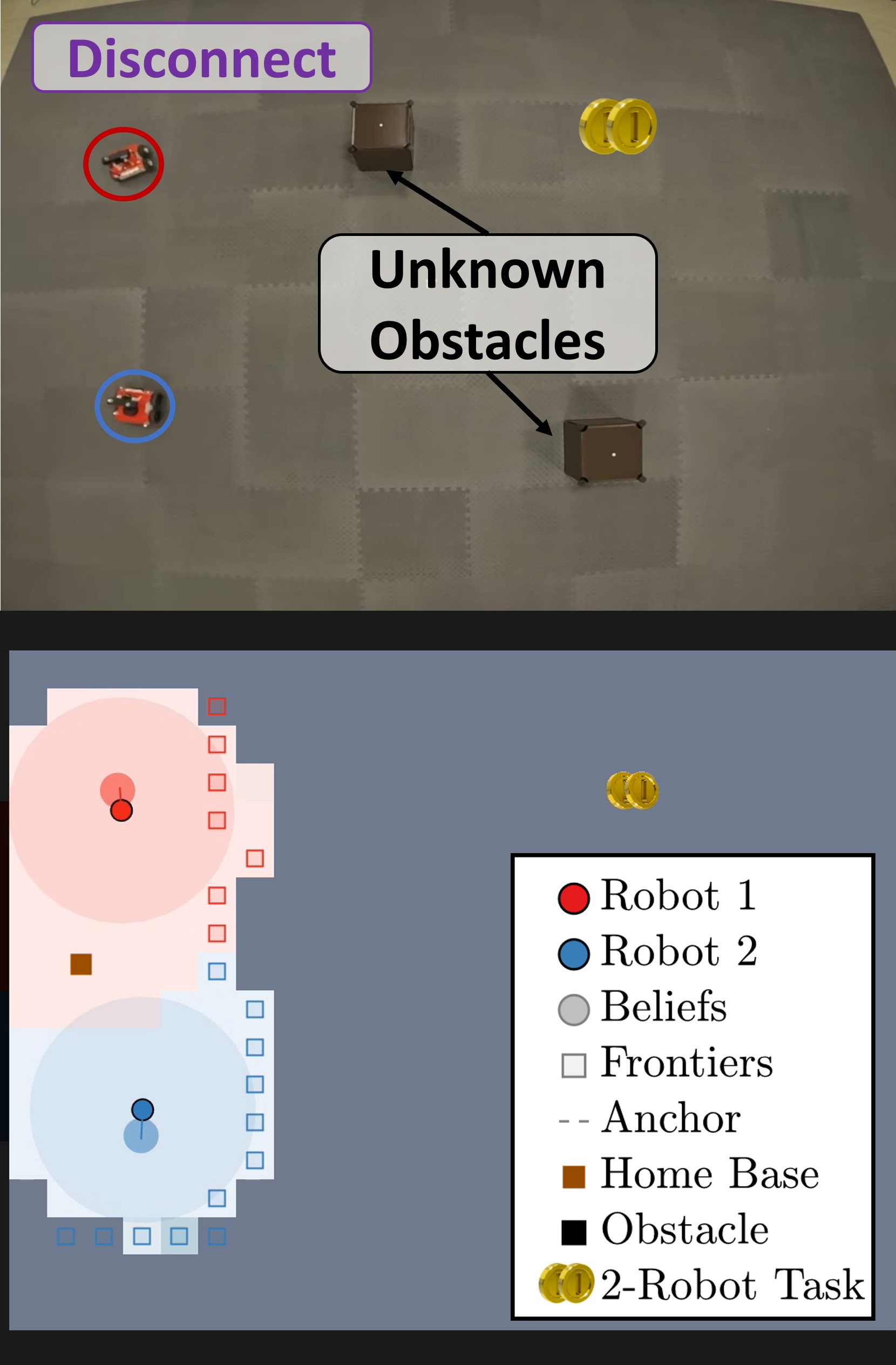

Supplement: Supplementary file 1 [file DataSheet1.ZIP › OrderedPictures/19Exp2Agents1TaskFinal0.jpg]

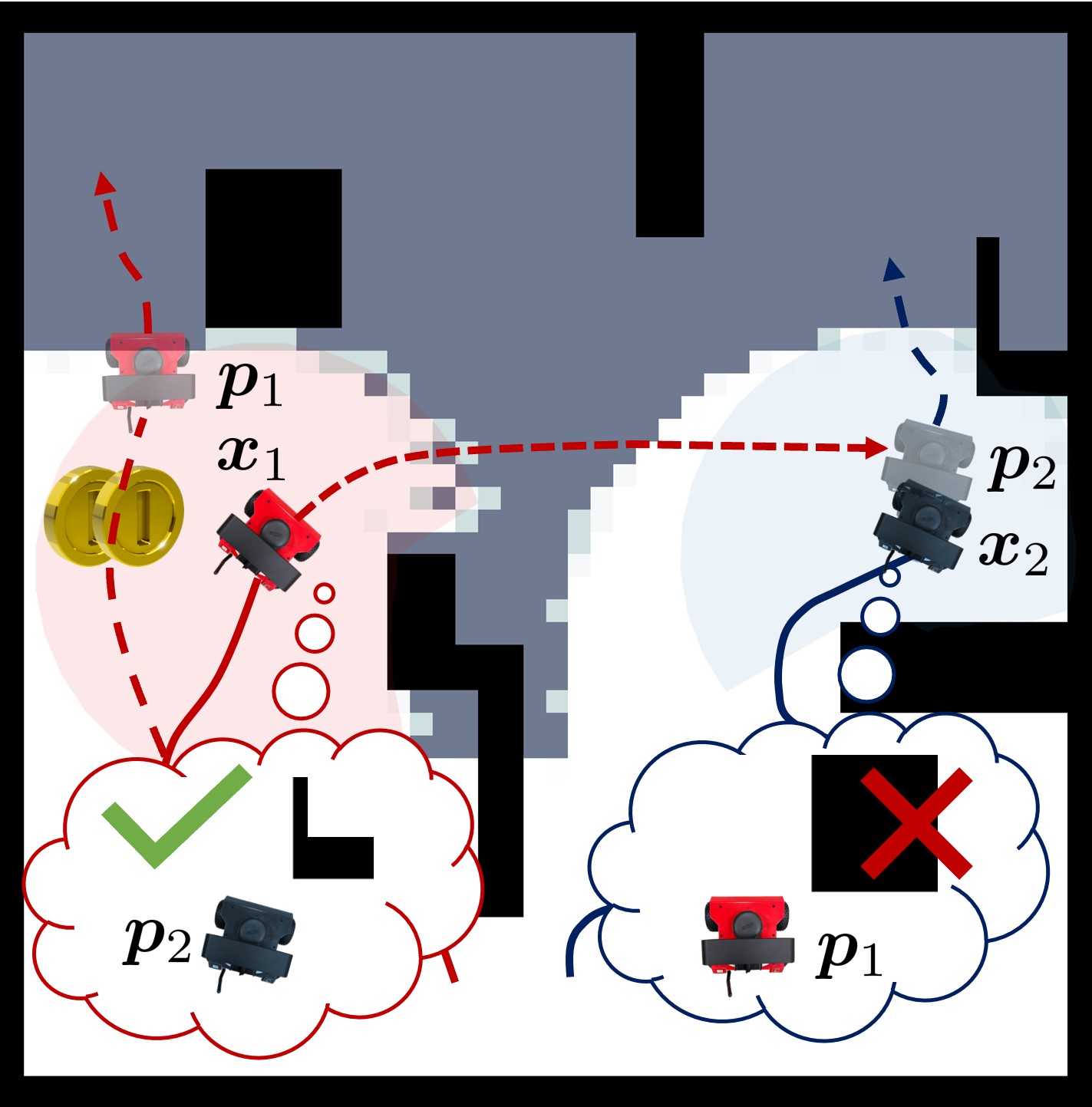

Supplement: Supplementary file 1 [file DataSheet1.ZIP › OrderedPictures/1IntroPic1.jpg]

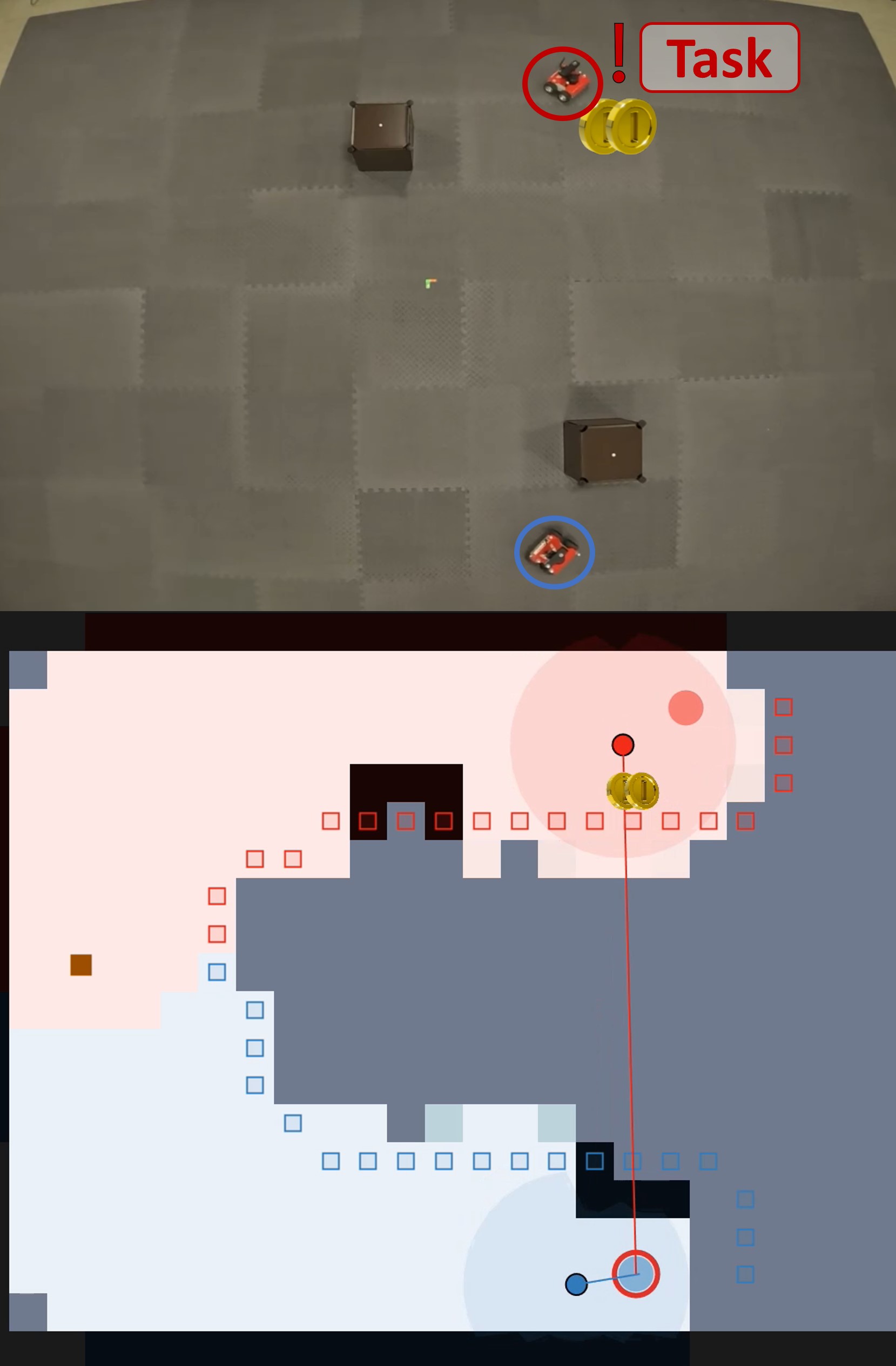

Supplement: Supplementary file 1 [file DataSheet1.ZIP › OrderedPictures/20Exp2Agents1TaskFinal1.jpg]

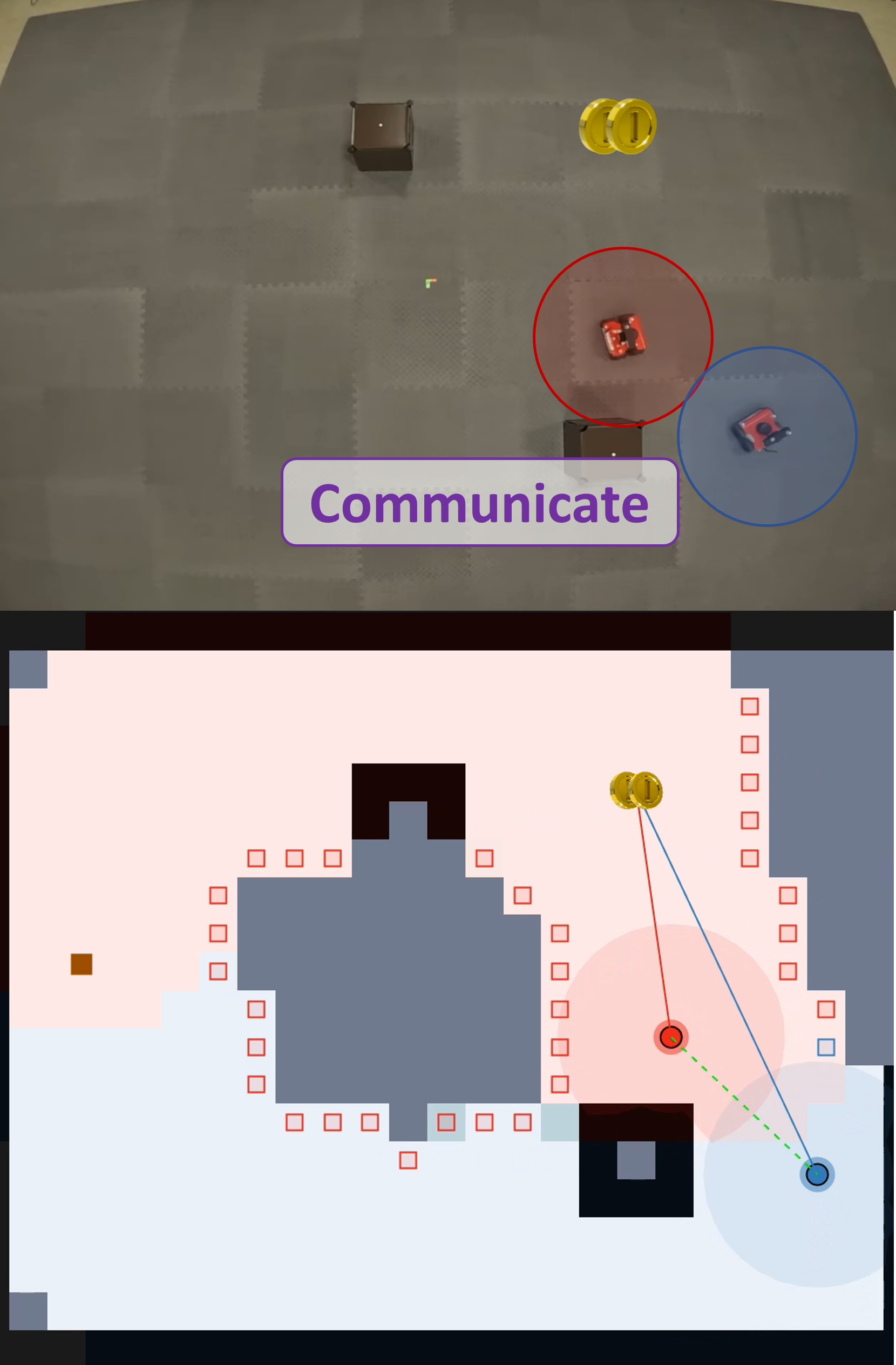

Supplement: Supplementary file 1 [file DataSheet1.ZIP › OrderedPictures/21Exp2Agents1TaskFinal2.jpg]

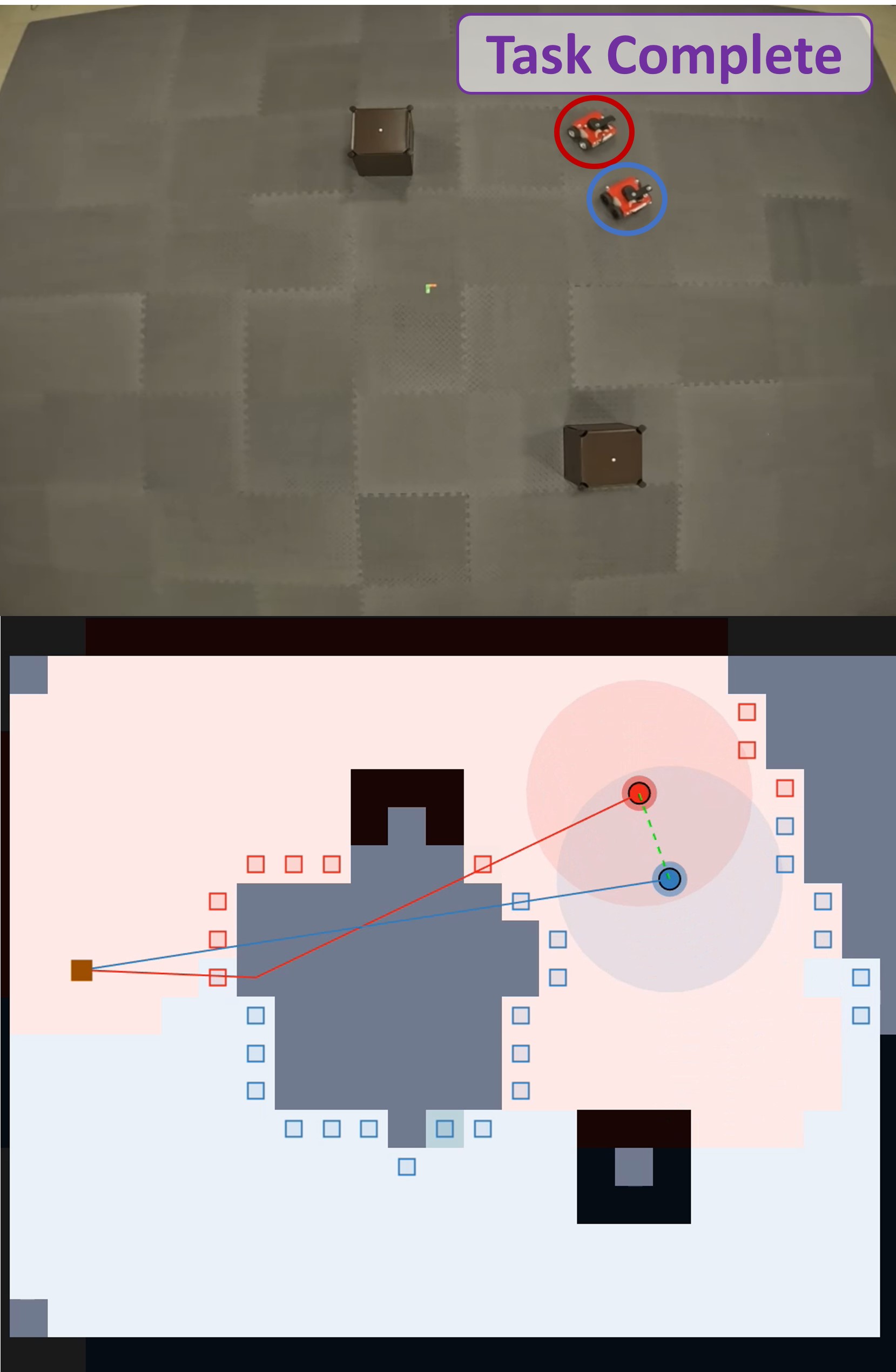

Supplement: Supplementary file 1 [file DataSheet1.ZIP › OrderedPictures/22Exp2Agents1TaskFinal3.jpg]

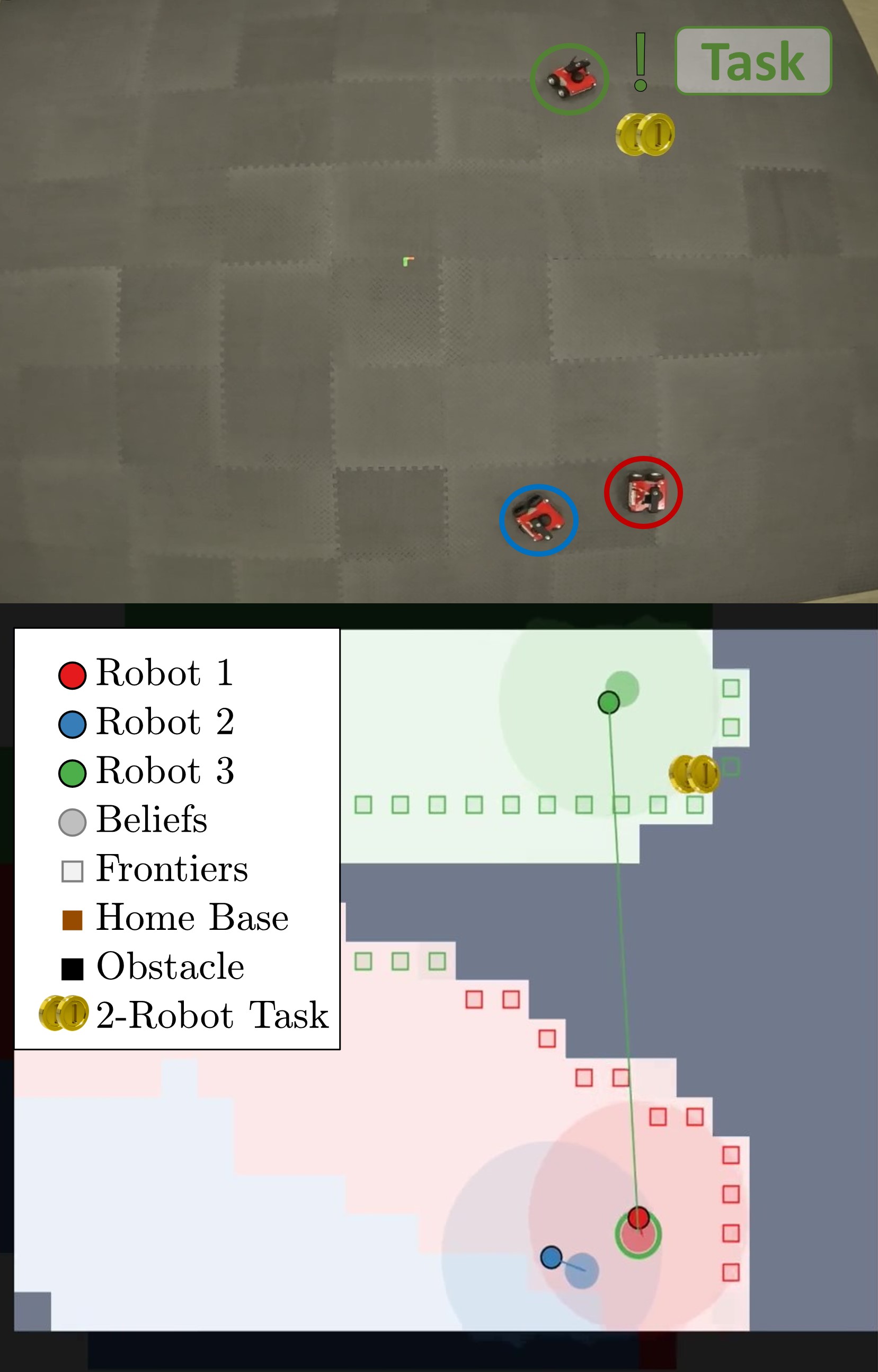

Supplement: Supplementary file 1 [file DataSheet1.ZIP › OrderedPictures/23Exp3Agents1TaskFinal0.jpg]

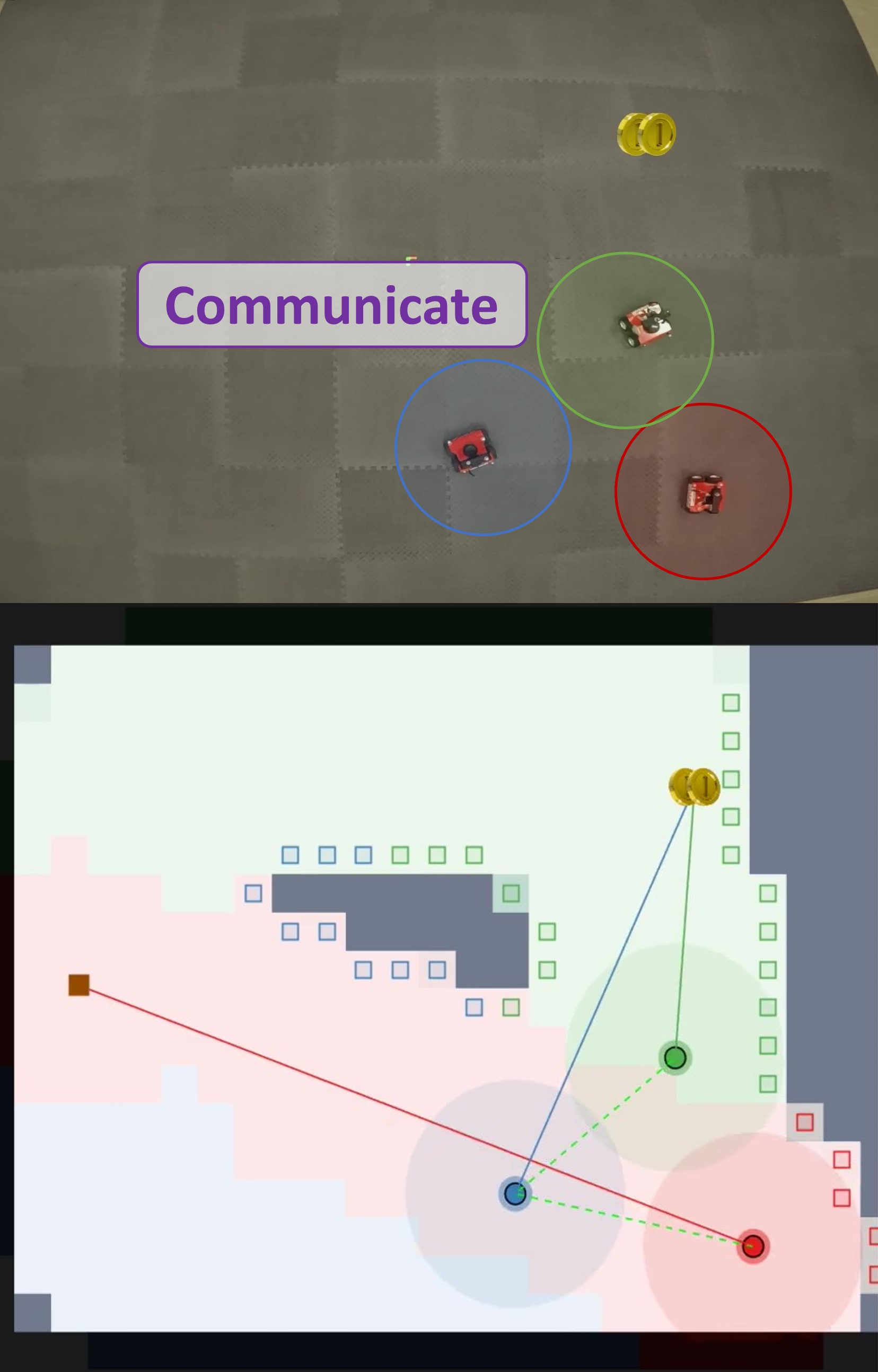

Supplement: Supplementary file 1 [file DataSheet1.ZIP › OrderedPictures/24Exp3Agents1TaskFinal1.jpg]

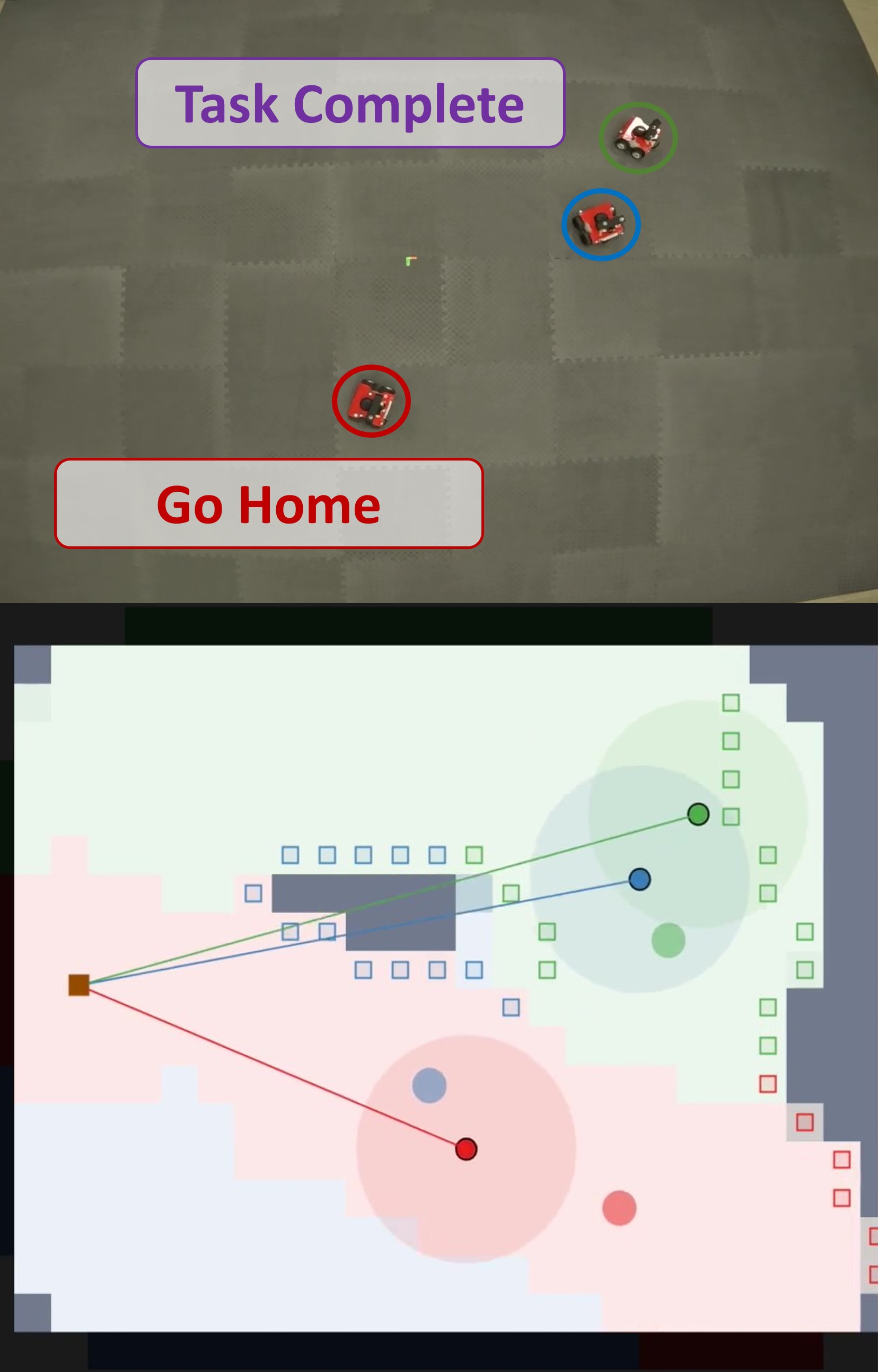

Supplement: Supplementary file 1 [file DataSheet1.ZIP › OrderedPictures/25Exp3Agents1TaskFinal2.jpg]

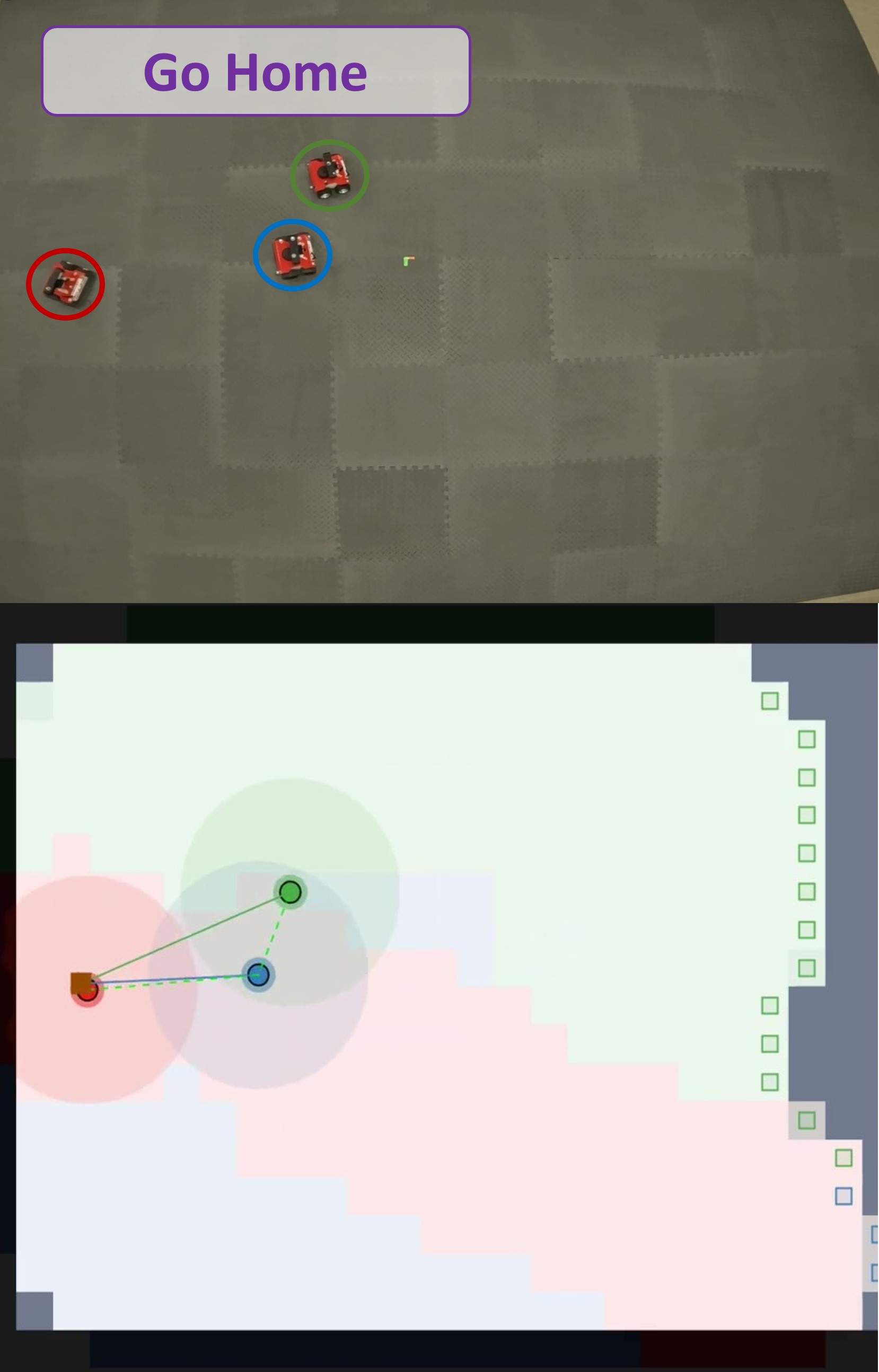

Supplement: Supplementary file 1 [file DataSheet1.ZIP › OrderedPictures/26Exp3Agents1TaskFinal3.jpg]

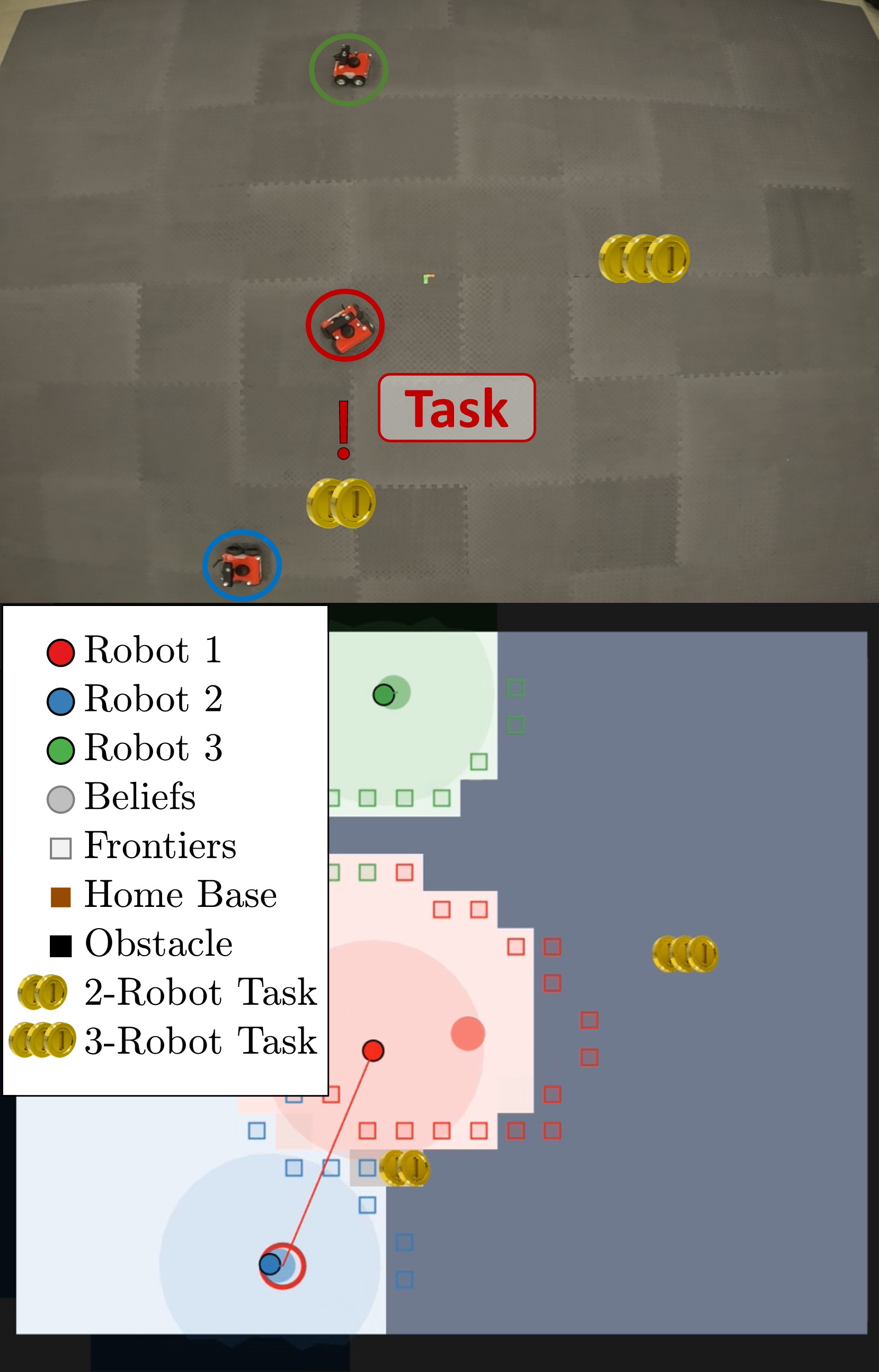

Supplement: Supplementary file 1 [file DataSheet1.ZIP › OrderedPictures/27Exp3Agents2TaskFinal0.jpg]

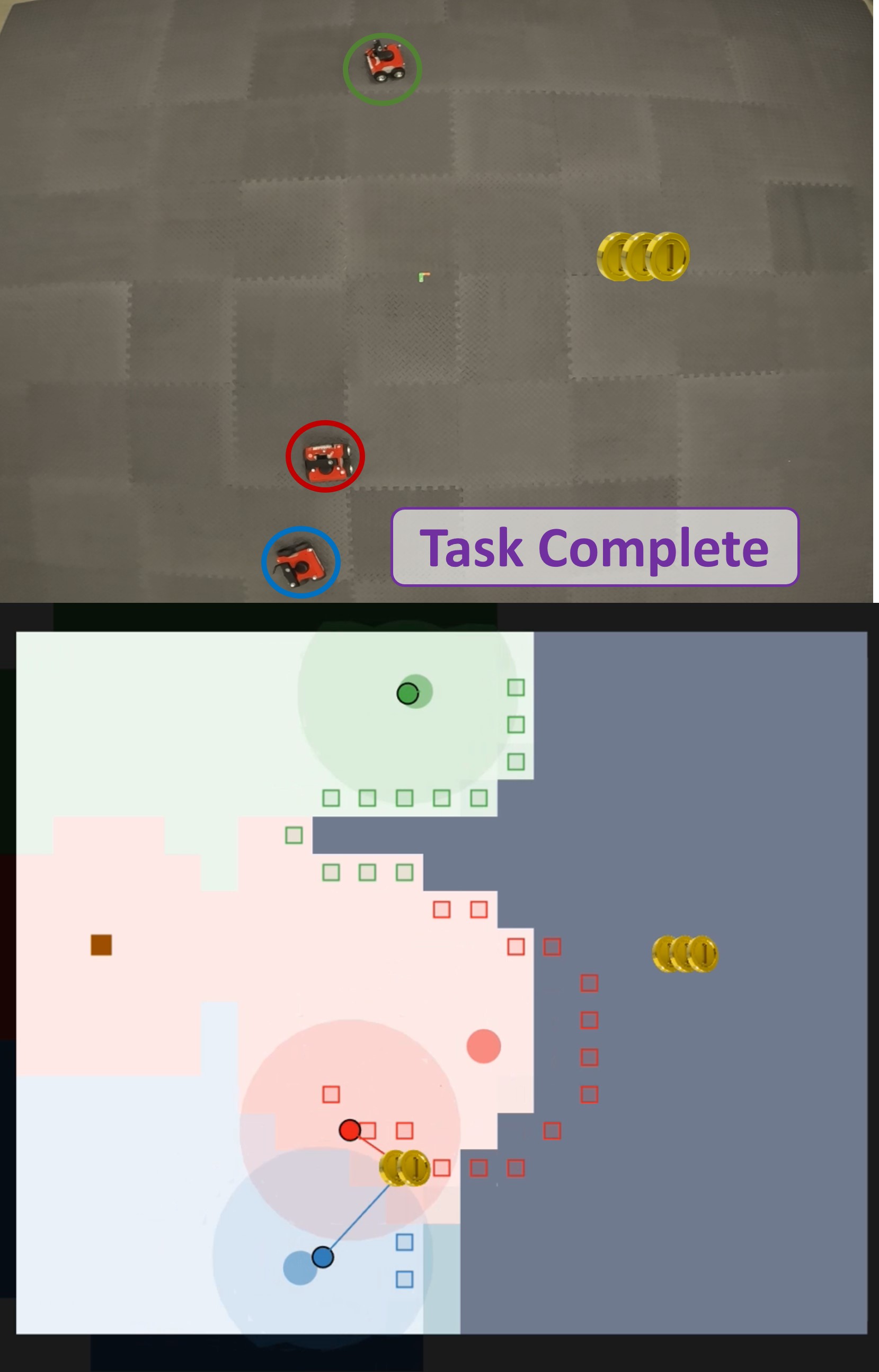

Supplement: Supplementary file 1 [file DataSheet1.ZIP › OrderedPictures/28Exp3Agents2TaskFinal1.jpg]

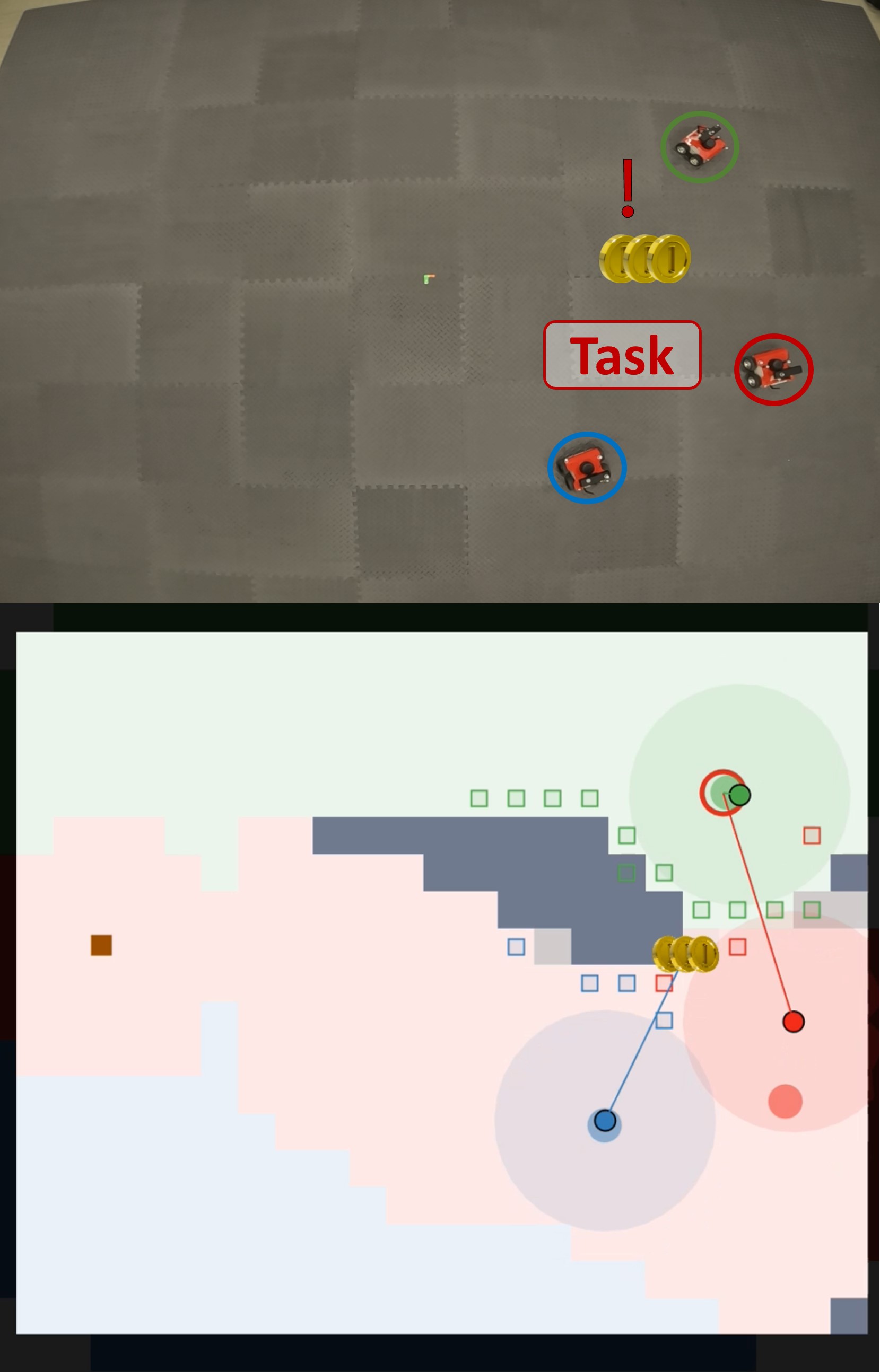

Supplement: Supplementary file 1 [file DataSheet1.ZIP › OrderedPictures/29Exp3Agents2TaskFinal2.jpg]

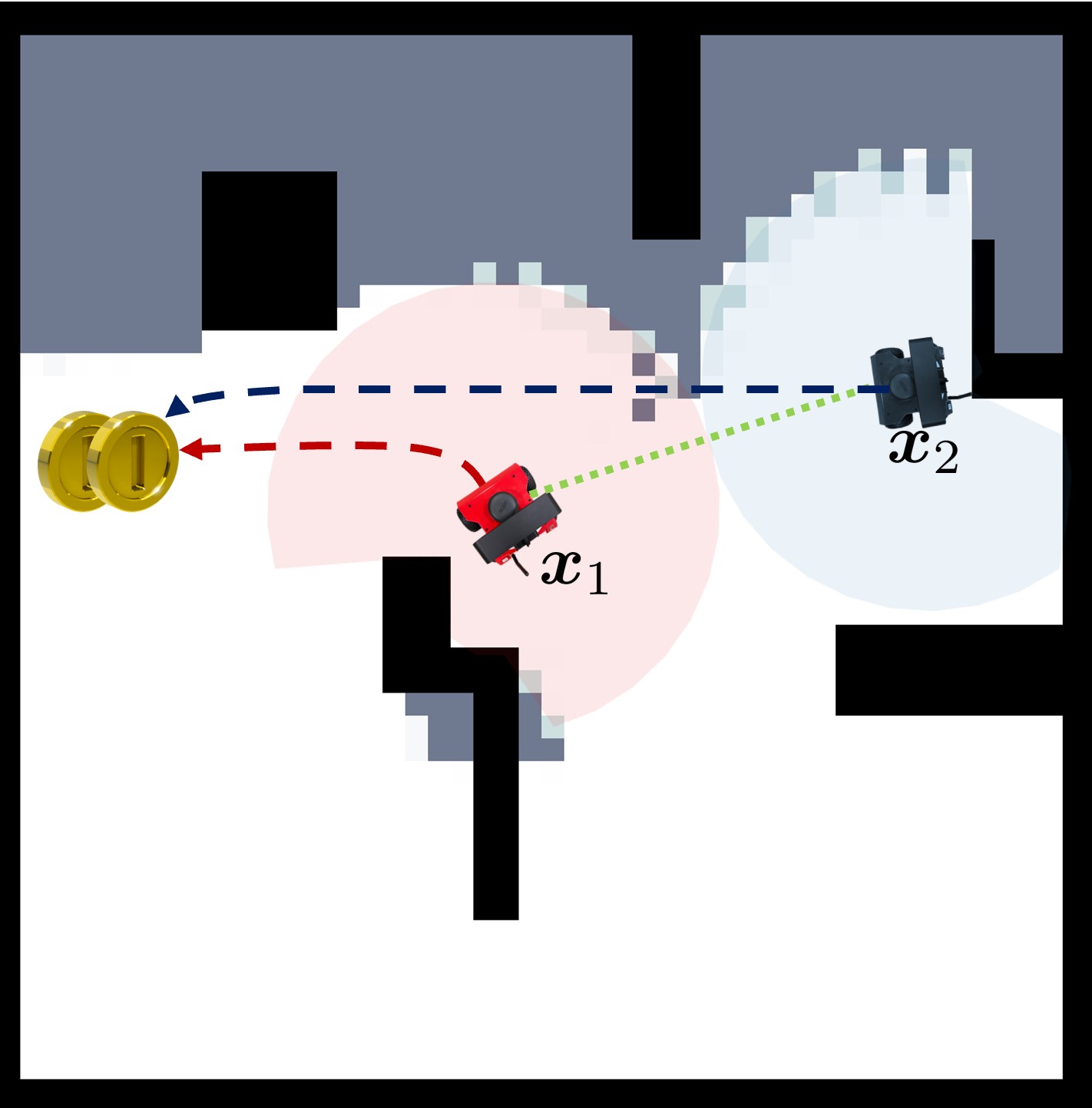

Supplement: Supplementary file 1 [file DataSheet1.ZIP › OrderedPictures/2IntroPic2.jpg]

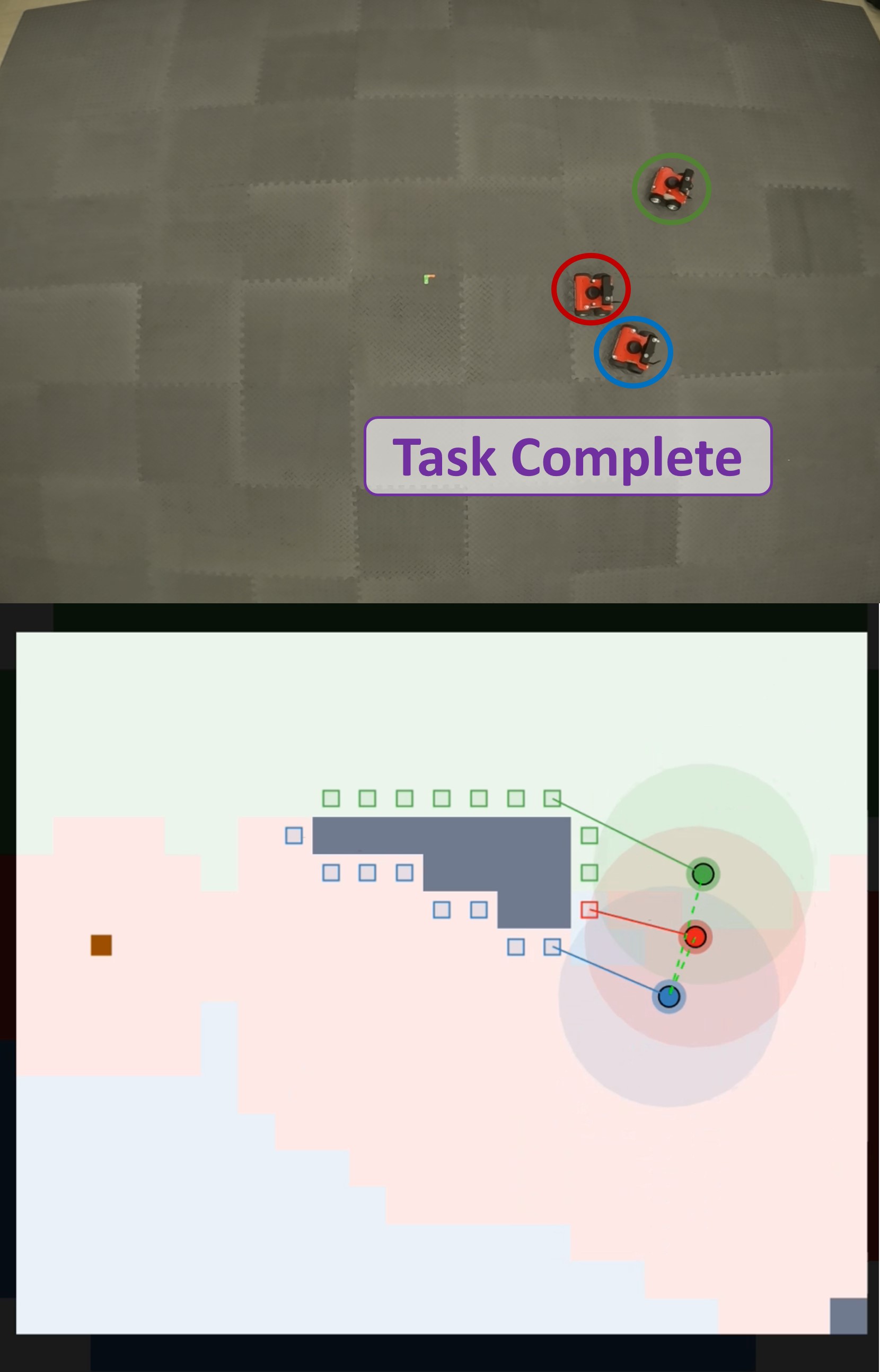

Supplement: Supplementary file 1 [file DataSheet1.ZIP › OrderedPictures/30Exp3Agents2TaskFinal3.jpg]

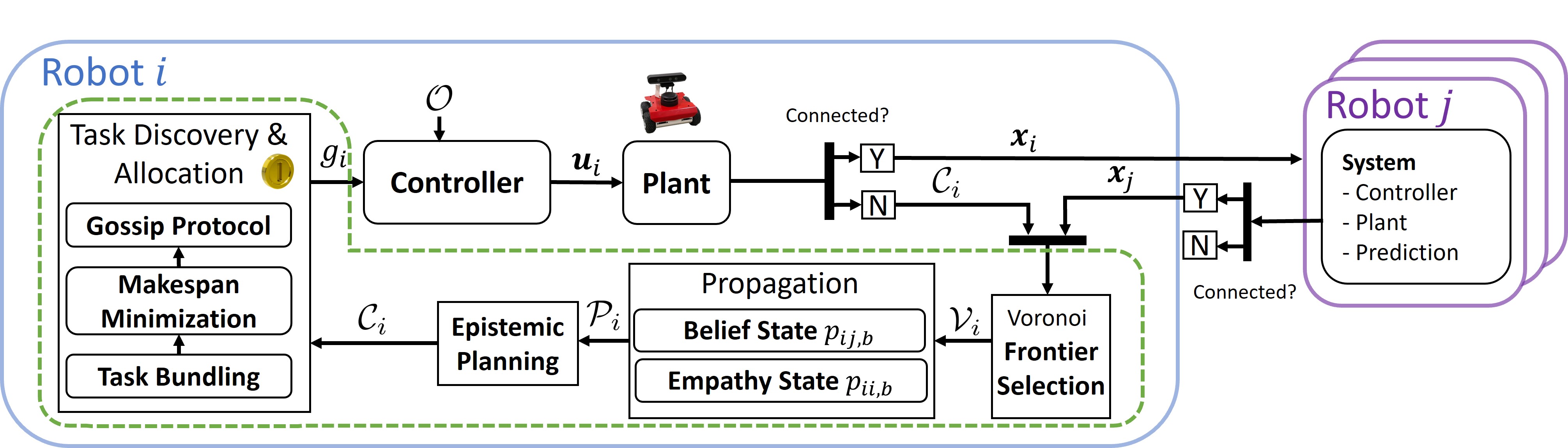

Supplement: Supplementary file 1 [file DataSheet1.ZIP › OrderedPictures/3ApproachFramework.jpg]

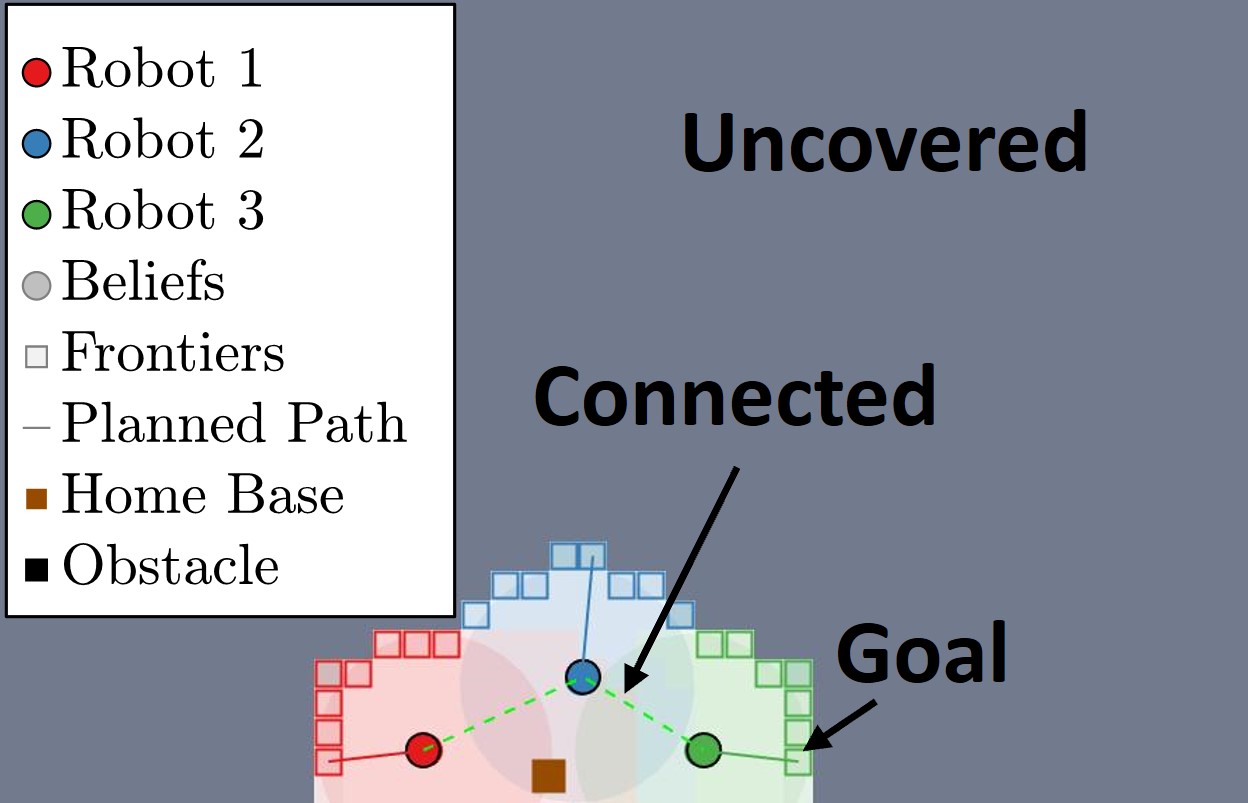

Supplement: Supplementary file 1 [file DataSheet1.ZIP › OrderedPictures/4coverage0.jpg]

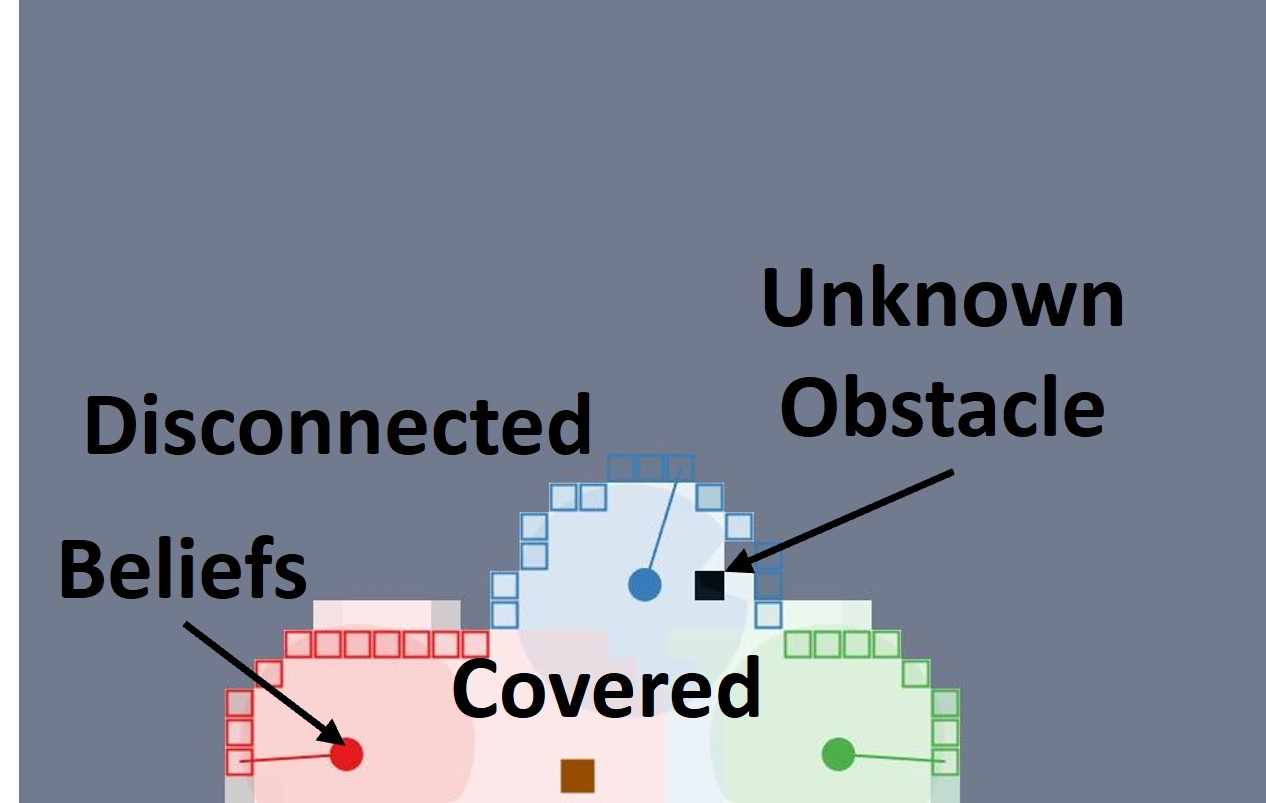

Supplement: Supplementary file 1 [file DataSheet1.ZIP › OrderedPictures/5coverage1.jpg]

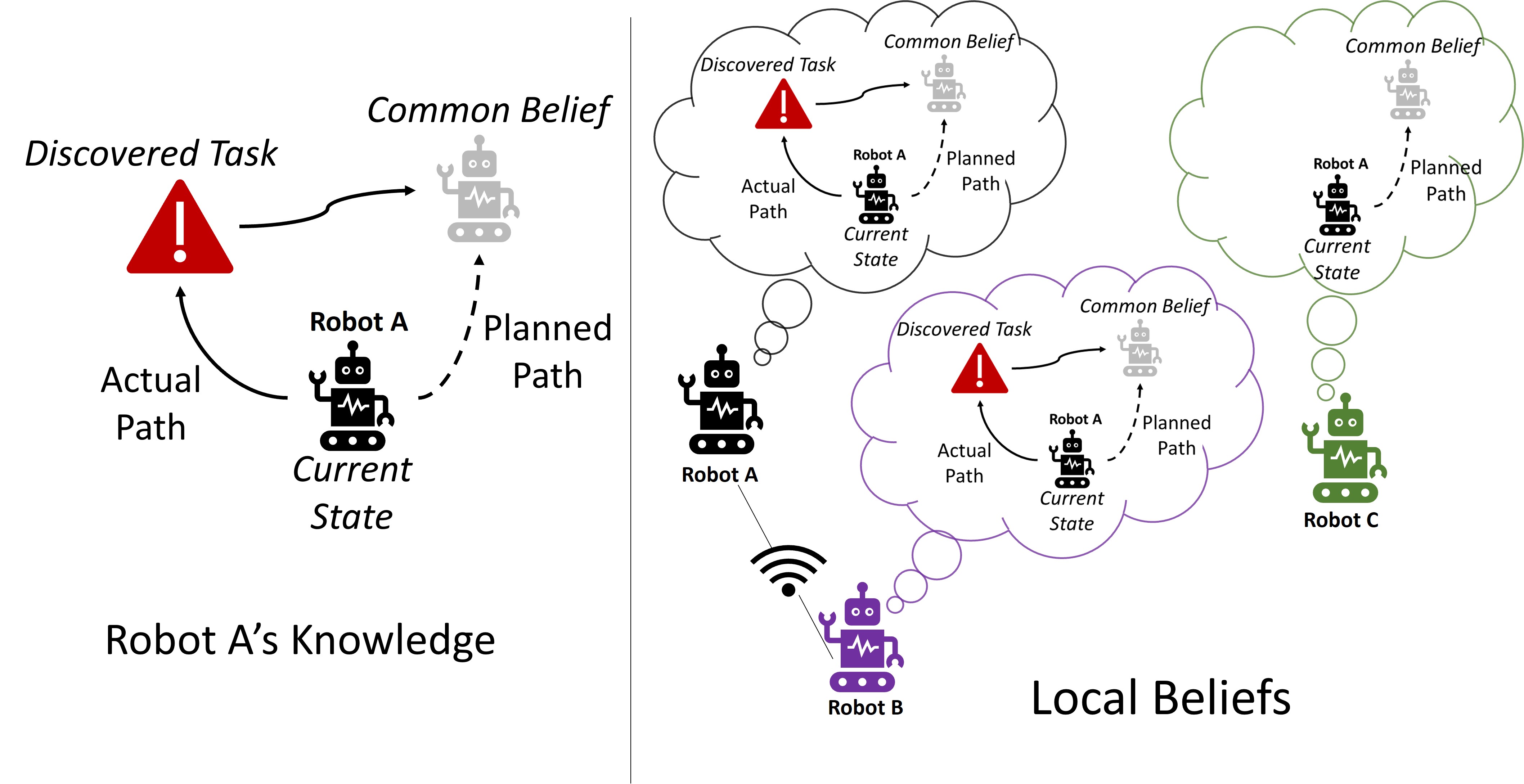

Supplement: Supplementary file 1 [file DataSheet1.ZIP › OrderedPictures/6epistemicPlanningFig2.jpg]

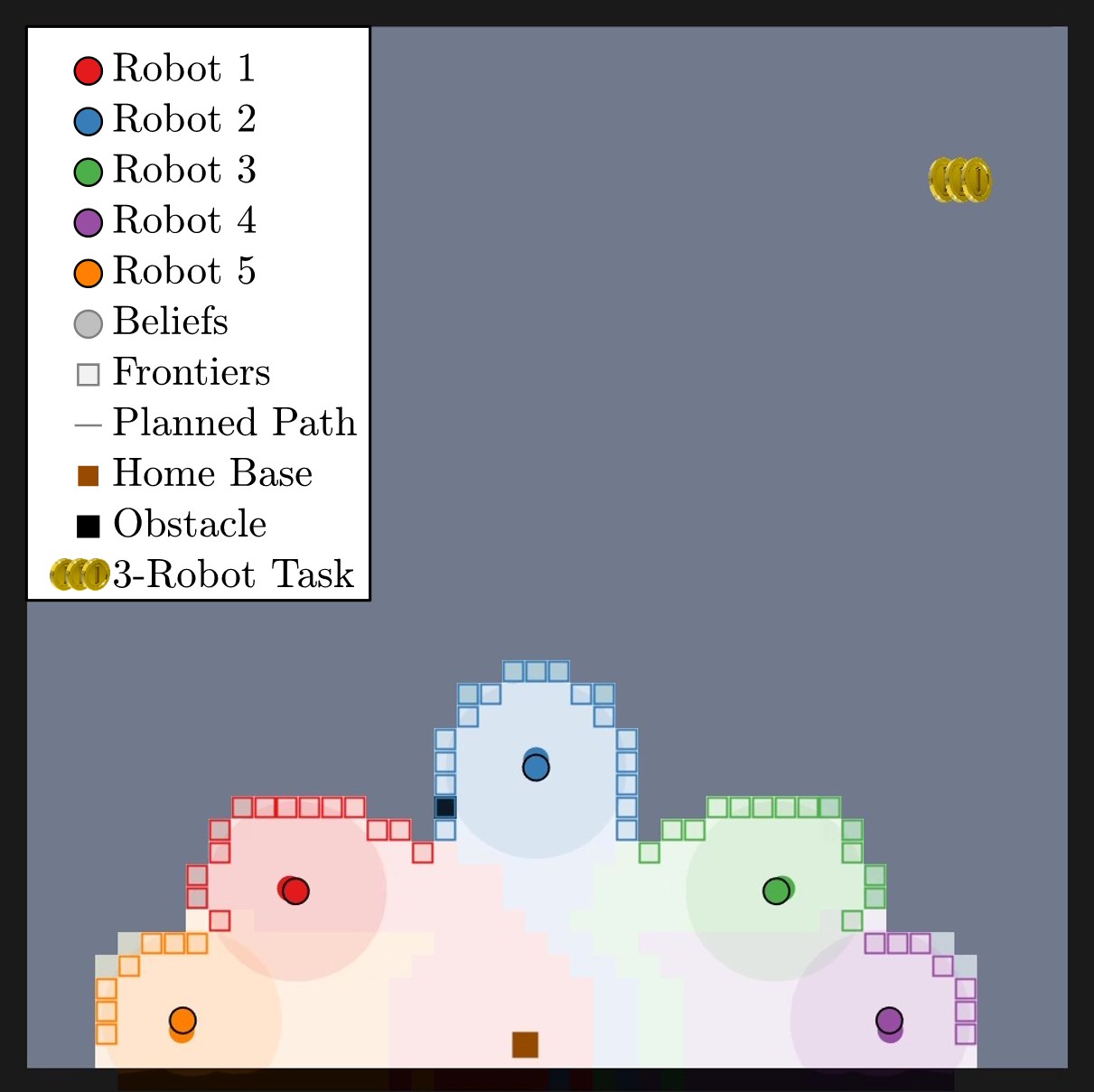

Supplement: Supplementary file 1 [file DataSheet1.ZIP › OrderedPictures/7MASTSimExamp0Final.jpg]

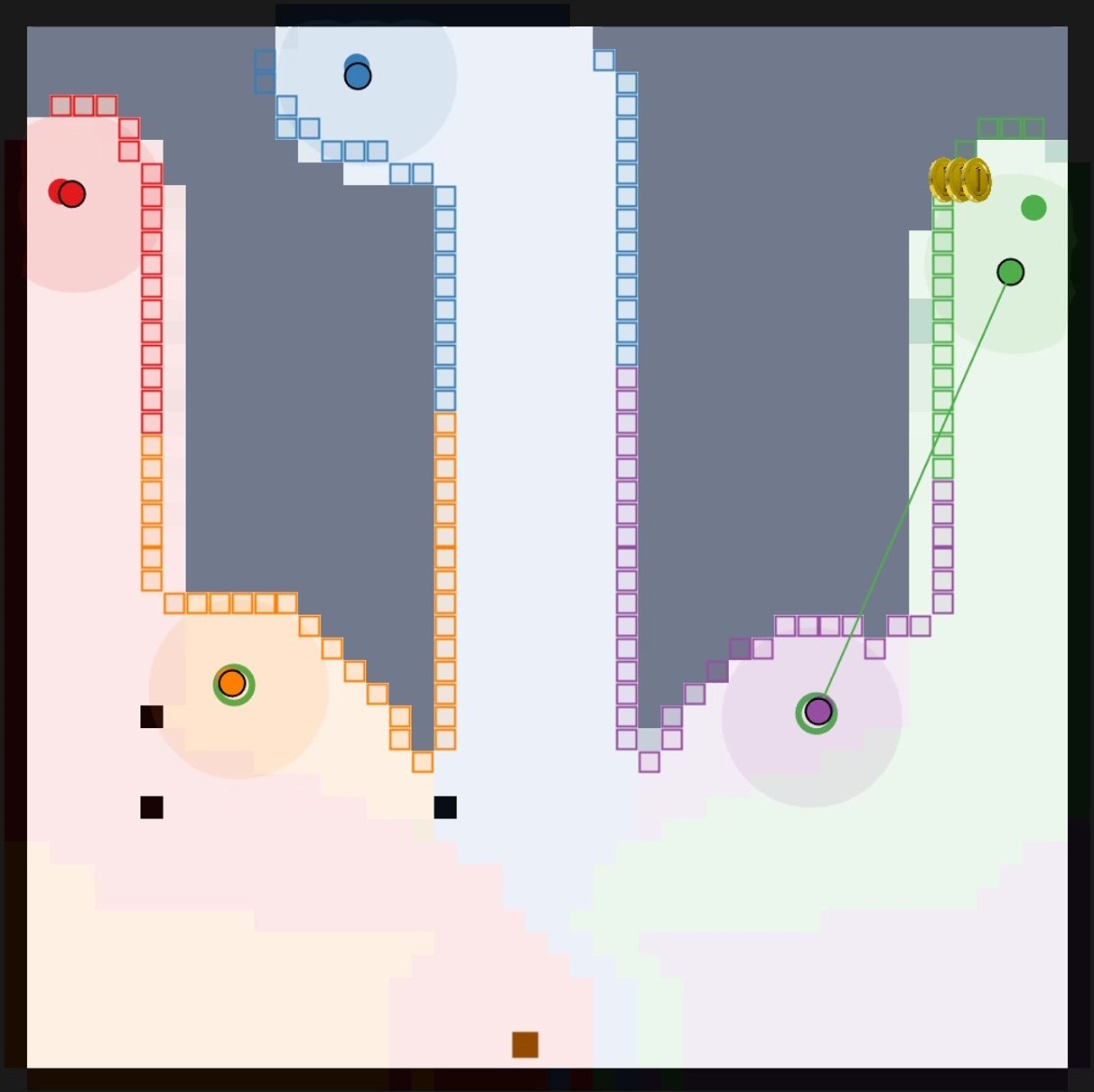

Supplement: Supplementary file 1 [file DataSheet1.ZIP › OrderedPictures/8MASTSimExamp1Final.jpg]

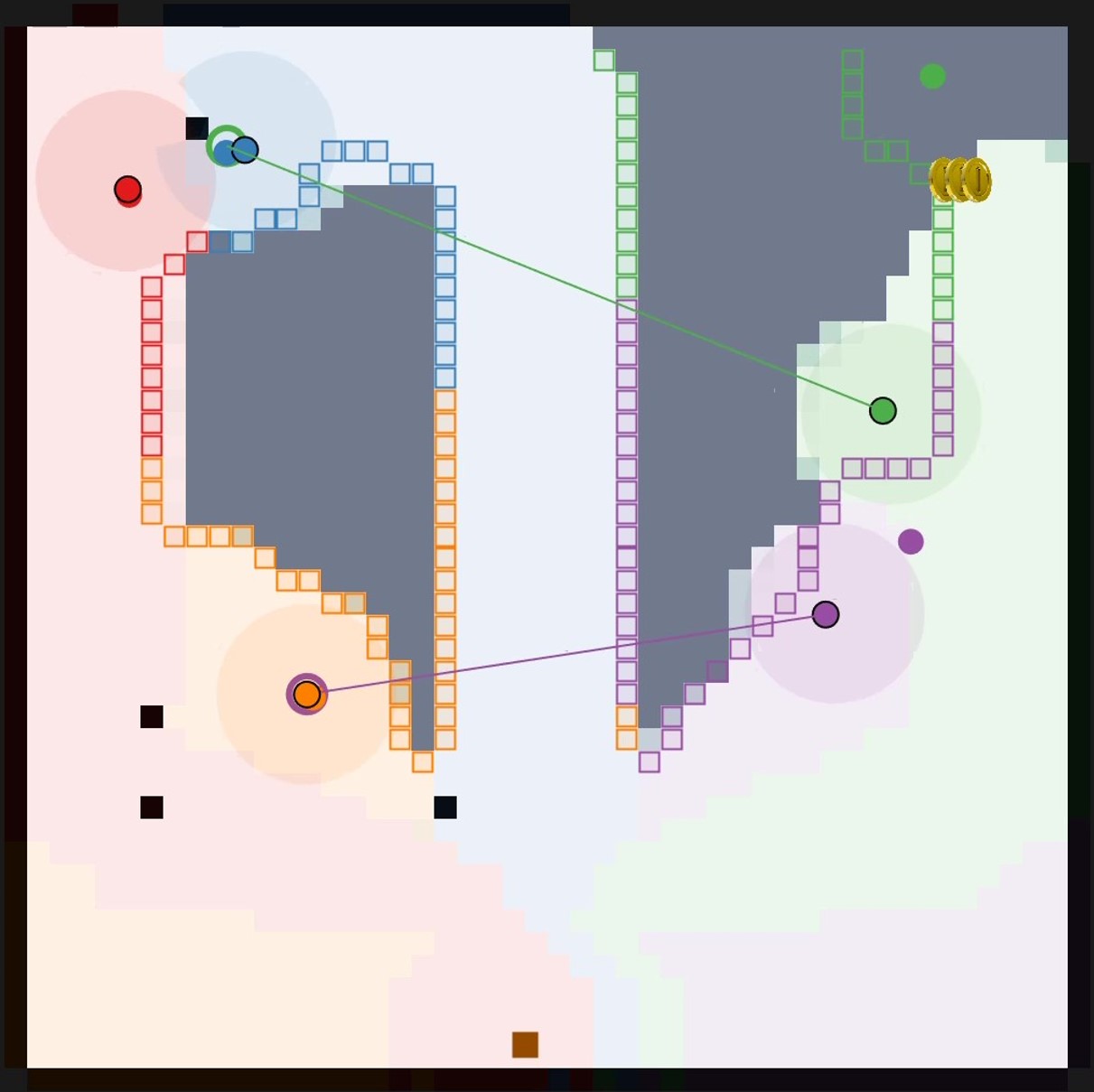

Supplement: Supplementary file 1 [file DataSheet1.ZIP › OrderedPictures/9MASTSimExamp2Final.jpg]
